# Transcriptomics of *Gabra4* knockout mice reveals common NMDAR pathways underlying autism, memory, and epilepsy

**DOI:** 10.1186/s13229-020-0318-9

**Published:** 2020-02-07

**Authors:** Cuixia Fan, Yue Gao, Guanmei Liang, Lang Huang, Jing Wang, Xiaoxue Yang, Yiwu Shi, Ursula C. Dräger, Mei Zhong, Tian-Ming Gao, Xinping Yang

**Affiliations:** 1 Department of Obstetrics and Gynecology, Nanfang Hospital, Southern Medical University, Guangzhou, 510515 China; 2 Institute of Neuroscience and Department of Neurology, The Second Affiliated Hospital of Guangzhou Medical University, Guangzhou, 510260 China; 3 Key Laboratory of Mental Health of the Ministry of Education, Southern Medical University, Guangzhou, 510515 China; 4 Department of Bioinformatics, School of Basic Medical Sciences, Southern Medical University, 1838 N. Guangzhou Ave, Guangzhou, 510515 China; 5 State Key Laboratory of Organ Failure Research, Guangdong-Hong Kong-Macao Greater Bay Area Center for Brain Science and Brain-Inspired Intelligence, Guangdong Key Laboratory of Psychiatric Disorders, Collaborative Innovation Center for Brain Science, Department of Neurobiology, School of Basic Medical Sciences, Southern Medical University, Guangzhou, 510515 China; 6 Department of Psychiatry, University of Massachusetts Medical School, Worcester, MA 01655 USA

**Keywords:** *Gabra4*, Autism, Epilepsy, Transcriptome, NMDARs, Interactome

## Abstract

Autism spectrum disorder (ASD) is a neuronal developmental disorder with impaired social interaction and communication, often with abnormal intelligence and comorbidity with epilepsy. Disturbances in synaptic transmission, including the GABAergic, glutamatergic, and serotonergic systems, are known to be involved in the pathogenesis of this disorder, yet we do not know if there is a common molecular mechanism. As mutations in the GABAergic receptor subunit gene *GABRA4* are reported in patients with ASD, we eliminated the *Gabra4* gene in mice and found that the *Gabra4* knockout mice showed autistic-like behavior, enhanced spatial memory, and attenuated susceptibility to pentylenetetrazol-induced seizures, a constellation of symptoms resembling human high-functioning autism. To search for potential molecular pathways involved in these phenotypes, we performed a hippocampal transcriptome profiling, constructed a hippocampal interactome network, and revealed an upregulation of the NMDAR system at the center of the converged pathways underlying high-functioning autism-like and anti-epilepsy phenotypes.

## Introduction

Autism spectrum disorder (ASDs) is a group of neurodevelopmental disorders with core clinical features of impaired social interaction, and communication withdrawal, stereotyped behaviors, and restricted interests [1, 2]. Individuals with autism show a wide range of variations in intelligence quotient (IQ); it can be normal, above average, or with intellectual disability. About 55% show intellectual disability (IQ < 70) [3] and 30% of children with autism may have severe intellectual disability [4]. ASD without an intellectual disability is called high-functioning autism (HF-ASD) [4]. About 20–30% of autistic children show symptoms of epilepsy [6, 7], whereas HF-ASD individuals have a lower incidence of epilepsy [8, 9]. These reports suggest that there might be an underlying relationship between autism, intelligence quotient, and epilepsy.

ASD shows high genetic heterogeneity. Targeted studies have identified several synaptic cell adhesion molecules such as neuroligins (*NLGN3*, *NL*GN4) [10], neurexins (*NRXN1* [11], *CNTNAP2* [12]), scaffolding proteins *SHANK2* [13] and *SHANK3* [14], and other molecules in synaptic transmission [15–17]. Genome scale genotyping technologies, such as microarray-based comparative genomic hybridization (CGH) and whole exome sequencing (WES), have detected a large number of genomic copy number variations (CNVs) [18], point mutations, and small insertion/deletions [19–21].

A general explanation for the high genetic heterogeneity despite similar phenotypic features is the hypothesis that the risk genes share a common molecular network [20, 22]. Together with our collaborators, we searched for common molecular mechanism by constructing a protein-protein interaction network for autism from 191 autism candidate genes by screening the whole human orfeome in 2014 [23]. Since then, the autism candidate genes have been increased dramatically because of genome-scale search for disease-associated genes. There are thousands of candidate genes now, yet we still do not know the common molecular mechanism. Therefore, it is imperative to identify the convergent pathways for this highly heterogenic disease. Recent transcriptome studies reveal quantitative differences in gene expression levels in postmortem brain tissues from autistic patients [24, 25], and identify some enriched pathways [26]. The transcriptomic analyses of brain tissues can provide insights into convergent molecular pathways in shared behavioral outcomes [26]. Due to the limitation of the availability of postmortem brain tissues from autism patients, different genetic mouse models for autism are essential for such studies.

As described above, some genetic variants in genes encoding for synaptic proteins [15–17, 27] are found to be causal to autism, pointing to the neurotransmission dysfunction as a potential underlying mechanism. The most common neurotransmitter systems involved in the pathogenesis of ASD are the GABAergic, glutamatergic, and serotonergic systems [28, 29], yet we do not know their connections at molecular level. We thought that, by knocking out the disease candidate genes in these three systems and looking for dysregulated pathways, we might be able to find the converged pathways linking these three neurotransmission systems to the different phenotypic domains of autism.

Dysfunction of GABAergic signaling in early embryo development can lead to autism [30]. γ-Aminobutyric acid (GABA) receptors were also reported to be downregulated in postmortem autistic brain samples [31]. Association studies also suggest that *GABRA4* and *GABRB1* contribute to the susceptibility for autism [32]. A family-based association and linkage disequilibrium study has found a genetic interaction between *GABRA4* and *GABRB1* in the etiology of autism [33]. Variations of *GABRA4* were found in ASD patients [33, 34]. The *GABRA4* is mainly expressed in the thalamus, striatum, cerebral cortex, dentate gyrus (DG), and CA1 region of hippocampus [35].

*GABRA4* is an essential subunit for extrasynaptic GABA receptor α4βδ GABA(A) receptors. Chandra et al. generated the first *Gabra4* knockout mouse model which exhibits a lack of tonic inhibition in DG cells and thalamic relay neurons, and is insensitive to the sedative and analgesic effects of an extrasynaptic GABA receptor agonist gaboxadol [35]. Three other studies show that *Gabra4* knockout mice restores synaptic plasticity and spatial learning during puberty [36], and the mice exhibit enhanced trace and contextual fear conditioning [37], and have larger dendritic spine and shaft profiles [38]. The GABAergic miniature inhibitory postsynaptic currents (mIPSCs) were reported to be decreased [35], while NMDA/AMPA conductance ratio [39] was reported to be increased. However, these previous *Gabra4* knockout mice were not evaluated for the autistic phenotypes, including the core features of autism such as impaired social interaction, stereotyped behaviors, or restricted interests. Here, in order to establish mutant *GABRA4* as a causal gene in autism and search for underlying mechanisms, we generated *Gabra4* knockout mice (*Gabra4*^−/−^) which showed core features of autism, enhanced spatial memory, and attenuated susceptibility to pentylenetetrazol-induced seizures. These phenotypes turn out to be similar to those of high-functioning autism. To search for the converging pathways underlying these phenotypes, we then performed hippocampal transcriptomics and interactomics studies and revealed a central position of the *N*-methyl-d-aspartate receptors (NMDARs) in the interconnected pathways linking autism, learning/memory, and epilepsy.

## Materials and methods

### Animal

The mice were housed under standard conditions (12 h/12 h light/dark cycle, access to dry food and water ad libitum). All the experimental procedures involving mice were approved by the Committee on Animal Care and Use at the Southern Medical University.

### Generation of *Gabra4* knockout mice

The generation of *Gabra4* knockout C57BL/6 mice by transcription activator-like (TAL) effector nucleases (TALEN) technology [40] was carried out in Cyagen Biosciences Inc. (China). Briefly, exon 1 of mouse *Gabra4* gene (GenBank accession number, NM_010251.2) was selected as target site, and TALEN mRNAs generated by in vitro transcription were then injected into fertilized eggs for KO mouse productions (Additional file 1: Figure S1A). The products were Sanger-sequenced to confirm the deletion. The details were provided in Supporting Information.

### Behavior analysis

All the tested subjects were 6–8-week-old male mice. Data were expressed as means ± SEM values and were assessed two-way analysis of variance (ANOVA) (Morris water maze test), unpaired two-tailed *t* test (three-chamber test, self-grooming test, marble buried test, open field test, elevated plus test, fear conditioning test, Y maze test, Morris water maze test) for comparisons using GraphPad Prism version 6 Software.

### Seizure susceptibility test

Pentylenetetrazol (PTZ, SIGMA) was dissolved in 0.9% saline and administered intraperitoneally to the wild-type and *Gabra4*^−/−^ mice (5–6-week-old males) at a dose of 60 mg/kg body weight in a total volume of 0.20–0.25 ml. The mice were monitored and video-recorded in a clear cage for 30 min. The video recordings were used to confirm the visual range of seizures. The behavioral indicators of seizure activity were as follows: (I) the first myoclonic twitch, (II) clonic convulsions for at least 5 s, (III) tonic hindlimb extension, and (IV) death [41].

### RNA preparation

For each genotype, three RNA samples were prepared. Each RNA sample was extracted from dissected hippocampi of three adult mice according to the manufacturer’s protocol (RNAeasy Mini Kit, Qiagen, USA). The quality and yield of the isolated RNAs was assessed using a NanoDrop Spectrophotometer (Thermo Fisher Scientific, Waltham, MA, USA) and Agilent 2100 Bioanalyzer (Agilent Technologies, Santa Clara, CA, USA). Only RNAs with a high RNA integrity number (RIN > 9) were selected and used for the subsequent sequencing.

### RNA-seq and differential expression analysis

RNA sequencing was performed at Berry Genomics (Beijing, China) using Illumina NovaSeq. Reference genome (mm10) and gene model annotation files were downloaded from UCSC Genome Browser. Reads numbers mapped to each gene were counted using HTseq-count (v0.9.0). Raw counts of genes with > 1 counts in at least four samples were used for the principle components analysis (PCA) by plotPCA DEseq2 function. Differential expression analysis on two groups was performed using the DESeq2 (v1.20.0) and the edgeR (v3.22.5). Differentially expressed genes (DEGs) were determined using a cutoff of adjusted *p* < 0.05 for DESeq2 and *p* < 0.01 for edgeR. Log2-transformed differential expressed genes data were used for expression heatmap by pheatmap1.0.10 package.

### Quantitative real-time PCR

Total mRNAs from hippocampal tissues were extracted using standard column purification according to the manufacturer’s instructions (RNAeasy Mini Kit, Qiagen, USA), and reverse transcription of RNAs into cDNA was performed using Evo M-MLV RT kit following the manufacturer’s instructions (Accurate Biotechnology Co. Ltd, China). Real-time PCRs were performed using Roche LightCycle 480II and SYBRR Green Realtime Master Mix (TOYOBO, Japan) following manufacturer’s instructions. All data were normalized to the expression of *Gapdh*. The relative expression level was calculated using the 2−∆∆CT method. The primers for qPCR were listed in Additional file 1.

### Co-immunoprecipitation

The hippocampal tissue was harvested in lysis buffer (Beyotime Biotechnology, China) with 1 mM PMSF. Tissue lysate was incubated on ice for 15 min and the debris was removed by centrifugation at 14,000 g for 15 min at 4 °C. A volume of 500 μl lysate was incubated with primary antibody or IgG antibody at 4 °C overnight, and 20 μl of protein A/G plus-Agarose (Santa Cruz, USA) was added into the mixture and then incubated on a rotating device for 3 h at 4 °C. The immunoprecipitates were collected by centrifugation at 1000 g for 5 min at 4 °C. The pellets were washed with 500 μl of lysis buffer (with 1 mM PMSF) for three times, boiled in protein loading buffer for 5 min, and then run on 8-12% SDS-PAGE gels and transferred to PVDF membrane (BioRad, USA). The membrane was blocked with 5% skim milk in TBST for 1 h at room temperature and incubated with primary antibody at 4 °C overnight. The membrane was washed three times (each time for 5 min) and then incubated with second antibody for 1 h at room temperature. Horseradish peroxidase (HRP) conjugated anti-IgG antibody was detected using Clarity Western ECL substrate (Bio-Rad, USA) with FluorChem E system (ProteinSimple, USA). The following primary antibodies were used: rabbit anti-GRIN1, rabbit anti-GluN2B, rabbit anti-PSEN1 (1:1,000, Cell Signaling Technology, USA). HRP-conjugated goat anti-rabbit IgG antibody and normal rabbit IgG antibody were purchased from Cell Signaling Technology.

### Functional analysis of differentially expressed genes

Functional annotations were done using Database for Annotation, Visualization and Integrated Discovery (DAVID) tools (v6.8) and terms were identified with false discovery rate (FDR) less than 0.05. Visualization and plot of top selected terms were done using ggplot2 package (v3.0.0). Enrichments were also performed for candidate genes of ASD, epilepsy, and schizophrenia. More details and data sources were provided in Supporting Information.

### Construction of hippocampal interactome, co-expression network, and DEG interactome

We constructed a hippocampal interactome by mapping 15,254 expressed genes from the mouse hippocampal transcriptome to the whole mouse interactome from Biological General Repository for Interaction Datasets (BioGRID). We calculated correlation coefficient using FPKM value (fragments per kilobase of transcript per million mapped reads) and constructed a co-expression network at a cutoff 0.75 of correlation coefficient using weighted correlation network analysis (WGCNA). We mapped the 1247 DEGs to the mouse hippocampal interactome to extract hippocampal DEG interactome containing these DEGs and their first neighbors if they have both physical interaction and co-expression relationship. For extraction of the networks, the self-loop edges and zero-degree nodes were removed. In order to control the extraction of the DEG-subnetwork from the protein-protein interaction (PPI) network, we shuffled the nodes of the PPI network for 1000 times followed by subnetwork extraction. Then the sizes of control subnetworks were plotted in comparison with the size of the real DEG subnetwork (Additional file 1: Figure S6A-B).

### Subnetworks for autism and epilepsy and learning/memory

We mapped the ASD candidate genes to the mouse hippocampal interactome to extract a subnetwork containing these candidate genes and their first neighbors if they have both physical interaction and co-expression relationship. The same method was also applied for extracting epilepsy (EP) and learning/memory (LM) subnetwork using their candidate genes as we described above. We performed the same network randomization to control the extraction of these subnetworks as described above. Then the sizes of control subnetworks were plotted in comparison with the sizes of the real ASD, LM, or EP subnetworks (Additional file 1: Figure S6C-H).

### Gene-pathway bipartite network analysis

The data of Kyoto Encyclopedia of Genes and Genomes (KEGG) Orthology of mouse were downloaded from KEGG database (https://www.genome.jp/kegg/pathway.html) to construct a mouse gene-pathway bipartite network. The hippocampal expressed genes of both wild-type (WT) and *Gabra4*^−/−^ mice were mapped to the network and the hippocampal gene-pathway bipartite network was extracted (Additional file 2: Table S17). The DEGs, ASD candidate genes, EP candidate genes, and LM-related genes (seed genes) were mapped to the gene-pathway bipartite network, and the pathways that were significantly enriched for seed genes (*p* < 10^−6^) and contained at least ten seed genes were kept. The gene-pathway bipartite subnetworks of DEGs, ASD candidate genes, EP candidate genes, and LM-related genes were obtained (Additional file 2: Table S18–21). The genes involved in each pathway of these bipartite subnetworks were shown in Additional file 2: Table S22–25 and NMDARs were highlighted. A converged gene-pathway network module shared by the ASD, EP, and LM gene-pathway bipartite subnetworks was extracted (Additional file 2: Table S26). The genes involved in each converged pathway were shown in Additional file 2: Table S27, and NMDARs were highlighted. The pathways enriched in DEG, ASD, EP, and LM gene-pathway bipartite subnetworks were compared with those enriched in corresponding PPI subnetworks (Additional file 2: Table S28–31).

### Electrophysiology

Mice 3–4 weeks old were used to electrophysiological recording. Whole-cell patch-clamp recordings of hippocampus pyramidal neurons were carried out as previously described [42]. The detail procedure is provided in Supporting Information.

## Results

### *Gabra4* knockout mice exhibit autistic-like behaviors, enhanced memory and attenuated susceptibility to pentylenetetrazol

We generated *Gabra4* knockout mice in the C57BL/6 strain by creating an 8-base deletion using TALEN technology. The genotypes of the mutant offspring were confirmed by Sanger sequencing of the PCR-amplified target region on the genomic DNA extracted from mouse pups (Additional file 1: Figure S1A).

**Fig. 1:**
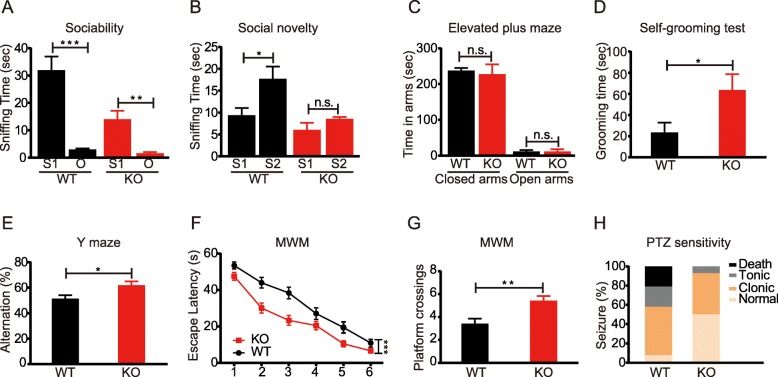
Autistic-like behaviors, enhanced learning/memory and attenuated susceptibility to pentylenetetrazol (PTZ) in *Gabra4*^−/−^ mice. **a** Both WT and *Gabra4*^−/−^ mice showed significant preference for stranger mice over objects (***p < 0.0001, **p = 0.0147. *n* = 16 for WT, and *n* = 8 for *Gabra4*^−/−^ mice, Student’s *t* test). **b** WT mice showed significant preference to novel mice over familiar mice (*p = 0.0042. *n* = 16 for WT, Student’s *t* test), but *Gabra4*^−/−^ mice did not show such preference (ns, *n* = 8, Student’s *t* test). **c** Compared to WT mice, *Gabra4*^−/−^ mice stayed the same time in both closed and open arms during the 5-min elevated plus maze test (*n* = 9 for WT, and *n* = 8 for Gabra4^−/−^ mice, *ns* no significance, Student’s *t* test). **d***Gabra4*^−/−^ mice spent more time to self-grooming than WT (*p = 0.0326, *n* = 10 for WT, and *n* = 9 for *Gabra4*^−/−^ mice, Student’s *t* test). **e***Gabra4*^−/−^ mice showed increased spontaneous alternation during Y maze test (*p = 0.0187, *n* = 12 for WT, and *n* = 9 for *Gabra4*^−/−^ mice, Student’s *t* test). **f** Escape latency of *Gabra4*^−/−^ mice in the Morris water maze (***p < 0.0001, *n* = 12 for WT mice, *n* = 16 for *Gabra4*^−/−^ mice. Two-way ANOVA test). **g** Number of platform crossings during probe trial in Morris water maze (**p = 0.0013, *n* = 12 for WT mice, *n* = 16 for *Gabra4*^−/−^ mice, Student’s *t* test). **h** Susceptibility to pentylenetetrazol in mice (In the test for 60 mg/kg PTZ, *p* = 0.0114, two-way ANOVA test)

We examined sociability and social novelty of the mutant mice in a three-chambered apparatus. During habituation to the empty chambers, neither wild-type (WT) nor *Gabra4*^−/−^ mice showed any preference for either left or right chamber (Additional file 1: Figure S1B). After habituation, both WT and *Gabra4*^−/−^ mice demonstrated a clear preference for the social stimulus over an empty cage, but WT mice showed better sociability based on the sniffing time to the stranger than to the object (Fig. 1a). But when a novel mouse was introduced into the previously empty cage, the WT mice preferred to sniff the novel mice, while the *Gabra4*^−/−^ mice did not show this preference (Fig. 1b), suggesting a deficit in social-novelty seeking in the mutant mice. These results demonstrated that the *Gabra4*^−/−^ mice exhibited reduced social interaction tendencies, which are characteristic features of autism. To exclude the possibility that social deficits might have resulted from locomotor dysfunction, we evaluated locomotor performance in the open field test and found that WT and *Gabra4*^−/−^ mice had no significant differences in the total distance traveled (Additional file 1: Figure S1C). This result indicated that both WT and *Gabra4*^−/−^ mice have normal locomotor activity. In the open field test, we also measured the center time during the first 5 min and found no significant difference between WT and mutant mice (Additional file 1: Figure S1D), suggesting that the mutant mice had no anxiety. In addition, we also tested for anxiety in the elevated plus maze and found no differences between the mutant and WT mice (Fig. 1c). We performed self-grooming test and found that *Gabra4*^−/−^ mice displayed more frequent grooming compared to WT mice (Fig. 1d), which is suggestive of stereotypical autistic behaviors. However, in the marble burying test, the *Gabra4*^−*/*−^ and WT mice buried approximately the same number of marbles in a 30-min test (Additional file 1: Figure S1E). Marble burying is commonly used to test for repetitive, anxiety, and compulsive-like behaviors [43], but the interpretation is controversial [44]. Considered together with the results from elevated plus maze (EPM) (Fig. 1c) and open field test (OFT) (Additional file 1: Figure S1D), the result of marble burying can be interpreted as no anxiety in mutant mice.

We evaluated the cognitive function of the mutant mice by performing Y-maze, fear conditioning, and Morris water maze tests. Compared to WT, the *Gabra4*^−*/*−^ mice showed a small but significant increase in their alternation percentage during Y-maze testing (Fig. 1e), suggesting that the mutant mice may have better spatial learning and memory. We also conducted fear conditioning test, and found that *Gabra4*^−*/*−^ mice showed normal fear memory (Additional file 1: Figure S1F-G). We further evaluated the spatial learning and memory ability by Morris water maze, assessing learning capacity via escape latency (i.e., time spent to reach the hidden platform). During the training trials, the mice in all groups showed significant improvement in escape latency time to find the submerged platform, but the *Gabra4*^−*/*−^ mice were significantly faster than the WT mice (Fig. 1f), although the swim velocity for the *Gabra4*^−*/*−^ mice was not significantly different compared with WT mice (Additional file 1: Figure S1H). *Gabra4*^−*/*−^ mice also had higher number of crossing over the platform position during reverse trials (Fig. 1g), confirming the enhanced memory ability.

ASDs are frequently comorbid with epilepsy [6, 7] and thus we tested the impact of the *Gabra4* knockout on susceptibility to the seizure-inducing drug pentylenetetrazol (PTZ). At a dose of 60 mg/kg body weight, we found that the percentages of PTZ-induced convulsions and tonic were significantly decreased in the *Gabra4*^−/−^ mice compared to the control mice (Fig. 1h). Over 92% (12/13) animals of WT mice exhibited at least one of all three phases of seizures and 38.46% (5/13) died; but only 53.80% (7/13) of *Gabra4*^−/−^ mice exhibited clonic and tonic phases of seizure, and none died. This result demonstrated that *Gabra4* elimination attenuates the susceptibility to PTZ-induced seizure.

We quantified the density of spines along 30 μm sections of dendrites in the hippocampus (Additional file 1: Figure S2A). *Gabra4*^−*/*−^ mice exhibited increased spine density (*p* = 0.0403) (Additional file 1: Figure S2B). The length and thickness of postsynaptic densities (PSDs) did not show significant change (Additional file 1: Figure S2D-E).

**Fig. 2:**
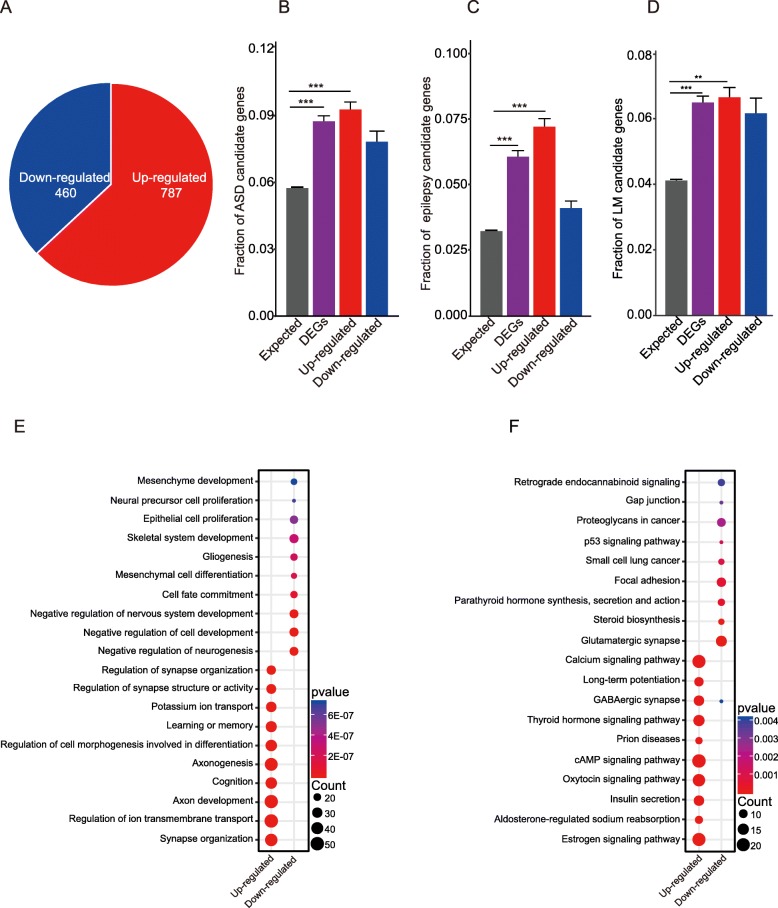
Hippocampal transcriptome sequencing analysis and functional annotation of differentially expressed genes. **a** Pie chart shows 787 up-regulated genes and 460 downregulated in *Gabra4* knockout mice (*n* = 3 samples per genotype). **b**–**d** Autism spectrum disorder **(b),** epilepsy **(c),** and learning/memory **(d)** candidate genes are enriched in *Gabra4*^−/−^ differential expression genes. ***p < 0.0001, Fisher’s exact test; Error bars represent the standard error of the fraction, estimated using bootstrapping method with 100 resamplings. **e**, **f** The top enriched biological processes of GO terms **(e)** and KEGG pathways **(f)** with upregulated genes and down-regulated genes. The enrichment analysis was performed using DAVID bioinformatics tool with a *p* value cutoff of 0.05 and FDR less than 0.05

We thought that hippocampal differentially expressed genes might be involved in autism and epilepsy. To systemically explore the molecular mechanism underlying autism and anti-epilepsy phenotypes, we performed transcriptome profiling on three mixed samples of hippocampal tissues from 12 adult *Gabra4*^−*/*−^ mice (each sample from four mice) and three mixed samples from 12 wild-type controls (each sample from four mice). The samples of *Gabra4* knockout mice were well separated from those of littermate WT mice in principle component analysis (PCA), which demonstrated that the samples from WT mice are good controls of the samples from the mutant mice (Additional file 1: Figure S3A). We detected a total number of 15,254 expressed genes (Additional file 2: Table S1), of which 1247 were differentially expressed genes (DEGs) (Additional file 2: Table S4), including 787 upregulated genes (Fig. 2a, Additional file 2: Table S5) and 460 downregulated genes (Fig. 2a, Additional file 2: Table S6). The 787 upregulated genes show significant enrichment with candidate genes for ASD (Fig. 2b), epilepsy (Fig. 2c), and learning/memory (Fig. 2d), suggesting that the upregulation of these genes may be involved in determining the autistic and anti-epilepsy phenotypes we observed in the mouse model. The upregulated genes also show significant enrichment with candidate genes of schizophrenia (Additional file 1: Figure S4A). The downregulated genes, however, did not show enrichments with the candidate genes for epilepsy, ASD, nor schizophrenia (Fig. 2b, c, Additional file 1: Figure S4A).

We performed functional enrichment analysis on the DEGs, and found that the upregulated genes and downregulated genes belong to distinct functional categories (Fig. 2e, f). The upregulated genes are enriched for functions in neuronal development and neuronal connections, which are shown in the enriched Gene Ontology (GO) terms (Fig. 2e, Additional file 1: Figure S4B-C): (i) biological processes such as axon development, synapse organization, ion transport and learning, or memory; (ii) cellular components such as axon part, distal axon, synaptic membrane, postsynaptic density, postsynaptic membrane, and presynapse; and (iii) molecular functions such as channel activity, metal ion transmembrane transporter activity, passive transmembrane transporter activity, and substrate-specific channel activity. Consistent with the enriched GO terms, the upregulated genes are enriched in pathways regulating neuronal development and synapse, such as cyclic adenosine monophosphate (cAMP) signaling pathway [45], calcium signaling pathway [46], and long-term potentiation [47] and GABAergic synapse (Fig. 2f). The downregulated genes are mainly enriched in the following GO terms (Fig. 2e, Additional file 1: Figure S4B-C): (i) negative regulation of nervous system development and neurogenesis; (ii) microtubule, proteinaceous extracellular matrix, extracellular matrix and extracellular matrix component; and (iii) negative regulation of neurogenesis, acidic amino acid transmembrane transporter activity, l-glutamate transmembrane transporter activity, and glycosaminoglycan binding. Consistent with enriched GO terms, the downregulated genes are enriched in pathways regulating synapse, such as focal adhesion [48], steroid biosynthesis [49], and glutamatergic synapse (Fig. 2f).

We checked the relative expression level of the genes involving inhibitory GABA and excitatory glutamate transmissions, including GABA receptors and Glutamate receptors (Additional file 1: Figure S3C-E). GABA A receptor alpha 2 (*Gabra2*) (Additional file 1: Figure S3C) and glutamate receptor *Grin1* was upregulated (Additional file 1: Figure S3D), but *Grin2c* downregulated (Additional file 1: Figure S3D). The expression levels of *Gabra2* and *Grin1* were confirmed by qRT-PCRs (Additional file 1: Figure S3F-G).

### Differential subnetwork enriched for genes involved in autism, epilepsy, and learning/memory

**Fig. 3:**
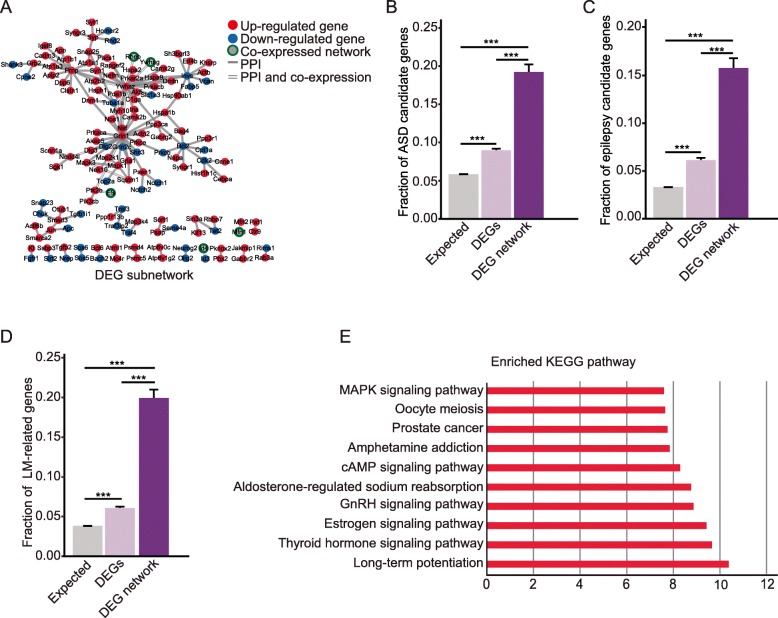
Hippocampal DEG interactome enriched with candidate genes involved in ASD, epilepsy and learning/memory. **a** The protein interaction network for DEGs (143 nodes and 145 edges). To extract the DEG subnetwork, a hippocampal interactome network was constructed by integrating the 15,254 hippocampally expressed genes and a protein interaction data from BIOGRID, and then the 1247 DEGs were mapped to the hippocampal interactome to extract a subnetwork including DEGs and their first co-expressed neighbors. Red node: upregulated; blue node: downregulated; gray node: without expression change; node with green border: co-expressed neighbor; gray line: protein-protein interaction (PPI); double lines: PPI and co-expression. **b** Enrichment of ASD candidate genes in DEG subnetwork compared to all DEGs. Fraction of ASD candidate genes = 0.0574 (876/15,254) in mouse expressed genes; 0.0874 (109/1247) in differentially expressed genes; 0.2028 (29/143) in DEGs in the mouse hippocampal interactome. *p* = 7.8167E-06 between expressed genes and DEGs; *p* = 4.1585E-06 between expressed genes and DEGs in the mouse hippocampal interactome; *p* = 2.0957E-09 between all DEGs and DEGs in the mouse hippocampal interactome. Error bars represent the standard error of the fraction, estimated using a bootstrapping method with 100 resamplings. **p < 0.01; ***p < 0.001 two-tailed fisher-exact test. **c** Enrichment of epilepsy candidate genes enriched in DEG subnetwork compared to all DEGs. Fraction of epilepsy candidate genes = 0.0325 (496/15,254) in expressed genes; 0.0609 (76/1247) in DEGs; 0.1608 (23/143) in DEGs in the mouse hippocampal interactome. *p* = 1.001E-07 between expressed genes and DEGs; *p* = 2.156E-10 between expressed genes and DEGs in the mouse hippocampal interactome; *p* = 3.7105E-06 between in all DEGs and DEGs in the mouse hippocampal interactome; Error bars represent the standard error of the fraction, estimated using a bootstrapping method with 100 resamplings. **p < 0.01, ***p < 0.001, two-tailed fisher-exact test. **d** Enrichment of LM-related genes in DEG subnetwork compared to all DEGs. Fraction of LM-related genes = 0.0384 (585/15,254) in expressed genes; 0.0626 (78/1247) in DEGs; 0.1958 (28/143) in DEGs in the hippocampal interactome. *p* = 1.443E-5 between expressed genes and DEGs; *p* = 8.1167E-13 between expressed genes and DEGs in the hippocampal interactome; *p* = 3.5114E-09 between all DEGs and DEGs in the hippocampal interactome. Error bars represent the standard error of the fraction, estimated using a bootstrapping method with 100 resamplings. **p < 0.01, ***p < 0.001, two-tailed fisher-exact test. **e** The top 10 KEGG pathways enriched with genes in the DEG subnetwork.

In order to search for a molecular network underlying the autistic-like and anti-epilepsy phenotypes, we first generated a mouse hippocampal interactome (Additional file 1: Figure S5), which contains 4204 nodes and 9205 edges by integrating the hippocampal expressed genes and protein interaction data from BioGRID, and then extracted DEG subnetwork containing these DEGs and their first neighbors if they have both physical interaction and co-expression relationship (Fig. 3a). The DEG subnetwork contains 143 nodes and 145 edges (Additional file 2: Table S7). We evaluated the involvement of the DEGs in the autistic behaviors, anti-epilepsy phenotype, and enhanced learning/memory by the enrichment analysis of the related genes. Compared to all DEGs, the DEG subnetwork exhibited a notable additional enrichment with both autism candidate genes (fraction of ASD candidate genes = 0.2028 (29/143)) (Fig. 3b), epilepsy candidate genes (fraction of epilepsy candidate genes = 0.1608 (23/143)) (Fig. 3c), and genes related to learning and memory (fraction of learning/memory candidate genes = 0.1958 (78/143)) (Fig. 3d).

The DEG subnetwork is enriched in pathways that are reported to be involved in autism and epilepsy, such as long-term potentiation [47], the cAMP signaling pathway [45], and mitogen-activated protein kinase (MAPK) signaling pathway [50] (Fig. 3e). These results suggest that the DEG subnetwork may contain pivotal pathways relevant for cognitive functions and perturbation of the molecular network may lead to autistic-like behaviors and neural synaptic activity related to epilepsy.

### Hippocampal interactome subnetworks for autism, epilepsy, and learning/memory

**Fig. 4:**
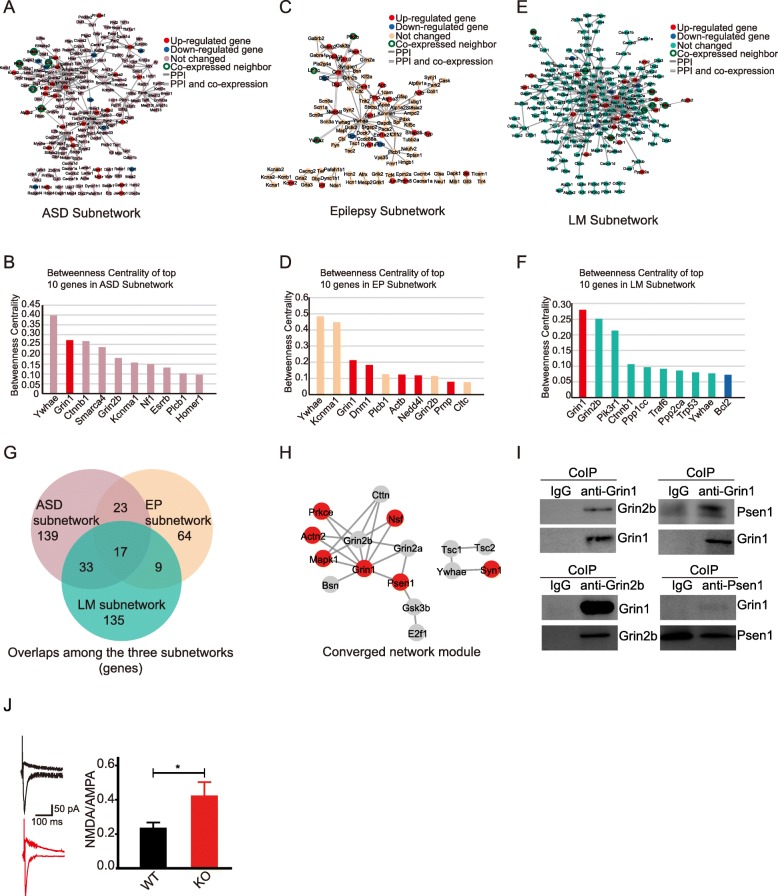
Hippocampal protein interaction subnetworks for ASD, epilepsy and learning/memory. **a** ASD subnetwork. ASD candidate genes were mapped onto the hippocampal interactome network to extract a subnetwork including ASD genes and first co-expressed PPI neighbors. **b** Network betweenness centrality of top 10 genes in ASD subnetwork (X-axis, top 10 genes; Y-axis, betweenness centrality). **c** EP subnetwork. Epilepsy candidate genes were mapped onto the hippocampal interactome network to extract a subnetwork including epilepsy genes and first co-expressed PPI neighbors. **d** Network betweenness centrality of top 10 genes in EP subnetwork (X-axis, top 10 genes; Y-axis, betweenness centrality). **e** LM subnetwork. Learning/memory (LM)-related genes were mapped onto the hippocampal interactome network to extract a subnetwork including LM-related genes and first co-expressed PPI neighbors. **f** Network betweenness centrality of top 10 genes in LM subnetwork (X-axis, top 10 genes; Y-axis, betweenness centrality). **g** Venn diagram of nodes in subnetworks. **h** The module of nodes shared by all three subnetworks. **i** Co-immunoprecipitation was performed on hippocampal tissue lysates from 8-week mice to detect interactions between endogenous protein pairs: GluN1 and GluN2B, GluN1 and PSEN1. Mouse IgG antibody was used as control in the pull down experiments. **j** Sample traces (left) and summary bar graph (right) of measurements of the ratio of NMDA receptor-mediated versus AMPA receptor-mediated synaptic responses recorded in slices; the NMDA/AMPA current ratio was determined by sequentially evaluating EPSC amplitudes at − 70 mV (AMPA) and at + 40 mV (NMDA) holding potential; NMDA receptor-mediated responses were measured with the mean response between 110 and 160 ms post-stimulus. All data presented as mean *±* SEM; *n* = 15 for WT cells from five mice and *n* = 14 for *Gabra4*^−/−^ cells from five mice; *p < 0.05, Student’s *t* test

We mapped ASDs candidate genes on the mouse hippocampal interactome (Additional file 1: Figure S5) and extracted a subnetwork of ASD genes and their first co-expressed PPI neighbors, which contains 212 nodes and 273 edges (Fig. 4a, Additional file 2: Table S8). Most of the genes in the subnetwork of ASD did not show expression change, with 13.7% (29/212) of the genes upregulated and 3.8% (8/212) downregulated. Some of these genes are well known to be involved in autism. For example, mutations in *Grin1* [51], *Myh10* [52, 53], *Mapk1* [54], and *Atp1a3* [55] were found in autism patients or mice. The expression change of these genes may perturb the subnetwork of autism, leading to autistic-like phenotypes of the knockout mice. To find out the key nodes that might have control over the autism subnetwork, we calculated the node betweenness centrality. The genes with the top 10 betweenness values were *Ywhaz*, *Grin1*, *Ctnnb1*, *Smarca4*, *Grin2b*, *Kcnma1*, *Nf1*, *Esrrb*, *Plcb1*, and *Hoer1* (Fig. 4b). Among these genes, only *Grin1* is differentially expressed (upregulated), suggesting it might play a key role in regulating the signaling network underlying autistic-like behavior of the knockout mice. Multiple studies have demonstrated that the NMDARs are involved in regulating synaptic plasticity [56] and the perturbations of NMDAR functions are found in autistic brain [57].

We mapped the epilepsy (EP) candidate genes on the mouse hippocampal interactome, and extracted a subnetwork of epilepsy candidate genes and their first co-expressed PPI neighbors (Additional file 1: Figure S5), which contains 113 nodes and 129 edges (Fig. 4c, Additional file 2: Table S10). Most of the genes (86/113) in the EP subnetwork did not show expression changes, with 24 genes upregulated, including *Grin1* (encoding the NMDAR subunit 1) and six NMDAR interactors (*Dlg3*, *Dnm1*, *Psen1*, *Ppp3ca*, *Myh10*, and *Ptk2b*), and three genes (*Dlg2*, *Dcx*, and *Flna*) being downregulated. The expression changes of these genes may perturb this subnetwork, and thus may be related to the anti-epilepsy phenotype. In order to identify the key genes that might have more control over the EP subnetwork, we calculated the node betweenness centrality for each of the nodes. Among the top 10 genes with the highest betweenness values (Fig. 4d), the genes *Grin1*, *Dnm1*, *Actb*, and *Prnp* are differentially expressed (upregulated). The dysregulated *Dnm1*, *Actb*, and *Prnp* interact with *Grin1*, suggesting that NMDAR system (NMDARs and their regulators) might contribute to the anti-epilepsy phenotype of this mouse model. We hypothesize that the upregulation of NMDAR interactors, such as *Dlg3*, *Myh10*, *Ppp3a*, *Psen1*, and *Dnm1*, may contribute to the anti-epilepsy phenotype by keeping the activity of NMDARs in control. This hypothesis is supported by the fact that mutations in *Dlg3* [58], *Psen1* [59], *Dnm1* [60], and *Ppp3ca* [61] have been found in epilepsy patients.

We mapped the 909 learning/memory (LM) related genes (Additional file 2: Table S11) to the mouse hippocampal interactome and extracted a LM subnetwork containing LM-related genes and their first co-expressed PPI neighbors, which contains 194 nodes and 343 edges (Fig. 4e, Additional file 2: Table S12). Most of the genes (167/194) in the LM subnetwork did not show expression changes, with 22 genes upregulated and five genes downregulated. Upregulated genes included *Grin1* and 21 other genes (Fig. 4e), and downregulated genes included five genes (*Shank3*, *Shc3*, *Grin2c*, *Prkcb*, *Bcl2*) (Fig. 4e). Interestingly, in the LM subnetwork, *Grin1* is one of the top 10 genes that have the biggest values of betweenness centrality (Fig. 4f), suggesting that they are located at the center of the subnetwork. *Grin1* may play a key role in regulating the signaling network through elevated expression. This result is consistent with previous findings that NMDARs are involved in synaptic plasticity, long-term potential, learning, and memory [27]. Interestingly, in both the autism, the EP and LM subnetworks, *Grin1* had the biggest betweenness values among DEGs (Fig. 4b, d, f), suggesting that NMDAR might play a key role in regulating molecular pathways underlying autism, anti-epilepsy, and enhanced learning/memory phenotypes.

The three subnetworks for autism, epilepsy, and learning/memory extracted from hippocampal interactome network are involved in different aspects of related brain functions. We compared the three subnetworks for their nodes (Fig. 4g) and found the overlapping part of the subnetworks (Fig. 4h). The overlapping part of these three subnetworks is a dense module, containing *Grin1* and *Grin2b* and their interactors *Prkce*, *Actin2*, *Mapk1*, *Bsn*, *Psen1*, *grin2a*, and *Nsf.* We carried out co-immunoprecipitation (Co-IP) for the endogenous proteins GRIN1, GRIN2B, and PSEN1 in mouse hippocampus. The GRIN1-GRIN2B and GRIN1-PSEN1 interactions were confirmed by Co-IP (Fig. 4i).

We speculated that the upregulated genes *Grin1* and *Prkce*, *Actin2*, *Mapk1*, *Psen1*, and *Nsf* might contribute to the comorbidity of autism with epilepsy and abnormal learning and memory. We measured the NMDA/AMPA conductance ratio recorded from the soma in whole-cell voltage clamp mode in response to stimulation of the Schaffer collateral pathway. Because NMDA EPSC is hard to quantify, we measured NMDA/AMPA conductance ratio as reported in a previous study [39]. Consistent with this study [39], we found an increased NMDA/AMPA conductance ratio in KO mice (Fig. 4j), suggesting the upregulated function of NMDARs compared with α-amino-3-hydroxy-5-methyl-4-isoxazole-propionic acid receptor (AMPAR).

### Converged pathways in subnetworks for autism, epilepsy, and learning/memory

**Fig. 5:**
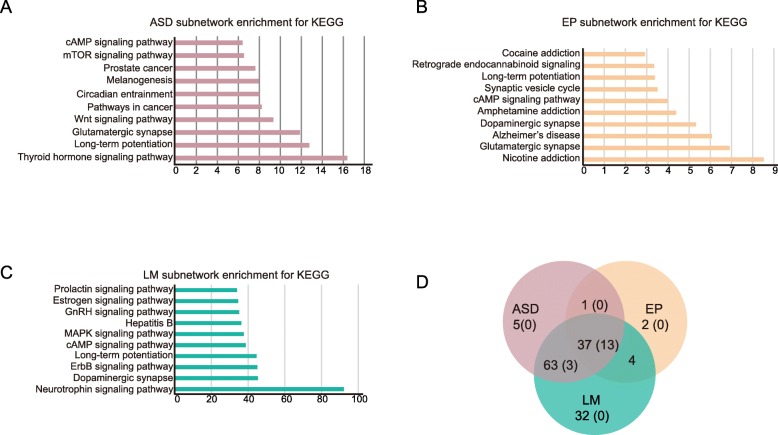
Enriched pathways with ASD, epilepsy, and LM subnetworks. **a** Top 10 enriched KEEG pathways with gene in ASD subnetwork. **b** Top 10 enriched KEGG pathways with genes in EP subnetwork. **c** Top 10 enriched KEGG pathways with genes in LM subnetwork. **d** The different and common enriched pathways among the three subnetworks. The numbers are enriched pathways with the subnetworks. The numbers in brackets are the pathways with NMDARs involved

To investigate potential shared pathways involved in autistic-like behaviors, resistance to epilepsy, and enhanced learning/memory, we performed functional enrichment analysis on the subnetworks for autism, epilepsy, and learning/memory (Additional file 1: Figure S7, Additional file 2: Table S16). The autism subnetwork is enriched in 101 KEGG pathways, many of which are well known involved in autism (Top 10 shown in Fig. 5a, full list in Additional file 2: Table S13), such as long-term potentiation [47], glutamatergic synapse [62], and Wnt signaling pathway [63, 64]. This subnetwork is also enriched in some pathways that are recently reported to be involved in autism, such as the circadian entrainment pathway [27, 65] and the thyroid hormone signaling pathway [66] (Fig. 5a, Additional file 2: Table S13). Of these 101 pathways, 16 directly involve NMDAR genes. The epilepsy subnetwork (EP subnetwork) is enriched in 44 KEGG pathways (top 10 shown in Fig. 5b and a full list in Additional file 2: Table S14). Some of them are known to be associated with epilepsy, such as long-term potentiation [67], synaptic vesicle cycle [68], cAMP signaling pathway [69], and glutamatergic synapse [70]. Of these 44 pathways, 13 directly involve NMDARs. LM subnetwork is enriched in 136 KEGG pathways (top 10 shown in Fig. 5c and full list in Additional file 2: Table S15). Some of them are known to be associated with learning and memory, such as long-term potentiation [47] and Dopaminergic synapse [48] signaling pathways. Of these 136 pathways, 16 directly involve NMDARs.

We further compared the enriched pathways of these three subnetworks and found 37 shared pathways (Fig. 5d), such as glutamatergic synapse, long-term potentiation, Wnt signaling pathway. Of the 37 shared pathways, 13 contain NMDARs (Additional file 2: Table S16). This observation is consistent with the converged molecular network module of the three subnetworks (Fig. 4h).

### Gene-pathway bipartite subnetworks for DEGs, autism, epilepsy, and learning/memory

We performed signaling network analyses using data from KEGG database (see Methods for details). The hippocampal gene-pathway bipartite network was extracted (Additional file 2: Table S17). Then we mapped DEGs, ASD candidate genes, EP candidate genes, and LM-related genes to the gene-pathway bipartite network and extracted gene-pathway bipartite subnetworks (Additional file 1: Figure S8A, C, E, G; Additional file 2: Table S18–25). We extracted a common network module from the DEG, ASD, EP, and LM gene-pathway bipartite subnetworks (Additional file 1: Figure S8I, Additional file 2: Table S26–27).

To validate the results obtained from PPI network analyses, we compared the results from signaling network analyses to those from PPI network analyses. The involved pathways in the DEG, ASD, EP, and LM gene-pathway bipartite subnetworks are largely overlapped with the pathways identified from PPI subnetworks (Additional file 1: Figure S8B, D, F, H, Additional file 2: Table S28–31). Of the 123 enriched pathways in DEG-pathway bipartite subnetwork, 86 (70.0%) of them are also found to be enriched pathways in DEG-PPI subnetwork (Additional file 1: Figure S8B, Additional file 2: Table S28). Of the 121 enriched pathways in ASD gene-pathway bipartite subnetwork, 82 (67.8%) of them are also enriched pathways in ASD PPI subnetwork (Additional file 1: Figure S8D, Additional file 2: Table S29). Of the 48 enriched pathways in EP gene-pathway bipartite subnetwork, 24 (50%) of them are enriched pathways in EP PPI subnetwork (Additional file 1: Figure S8F, Additional file 2: Table S30). Of the 183 enriched pathways in LM gene-pathway bipartite subnetwork, 132 (72.1%) of them are also found in LM PPI subnetwork (Additional file 1: Figure S8H, Additional file 2: Table S31). Consistent with the converged network module of the PPI subnetworks for autism, epilepsy, and learning/memory (Fig. 5h), the shared module of these three gene-pathway bipartite subnetworks also involve NMDARs (*Grin1*, *Grin2a*, and *Grin2b*) as hubs connecting to 16 pathways (Additional file 1: Figure S8 I and Additional file 2: Table S31).

## Discussion

The *GABRA4* gene was reported to be associated with autism in multiple ethnic groups [32, 33]. However, the molecular mechanism remains unclear. Previous studies on *Gabra4* knockout mice demonstrated the involvement of the *Gabra4* subunit in synaptic plasticity and spatial learning during puberty [36], and in contextual fear memory [37], but did not report any autistic-like behaviors [36, 37], probably due to the ignorance of its association with human autism. To investigate the causality of *GABRA4* in autism patients and underlying molecular mechanism, we generated knockout mouse model for *Gabra4* and evaluated their autistic-like behaviors, learning/memory abilities, and susceptibility to seizures, the three major phenotypic domains in autism spectrum disorder.

The three phenotypic domains of human autism include (1) core clinical features, such as impaired social interaction, and communication withdrawal, stereotyped behaviors and restricted interests [1, 2]; (2) abnormal intelligence [3, ﻿4﻿], such as severe intellectual disability (IQ < 50), intellectual disability (IQ < 70) and high-functioning autism (IQ > 70.); (3) relationship between epilepsy and IQ of patients: 34% prevalence of epilepsy in patients with IQ < 50, 27% in patients with IQ < 70, 9% in patients with IQ > 70, and 0.76% in normal population [8, 9, 71]. The core clinical features are shared phenotypes for all autistic patients, while the abnormal intelligence and epilepsy only appear in ASD subtypes. There are some relationships between autism and intellectual disability, and between autism and epilepsy [9]: lower IQ accompanies higher prevalence of epilepsy. Therefore, we tested the phenotypes belong to these three phenotypic domains in this potential animal model for HF autism.

The *Gabra4*^*−/−*^ mice displayed some autistic-like neurobehavioral dysfunction, specifically, impaired social interaction as manifested by a lack of preference for social novelty (Fig. 1b), and repetitive behavior evident as increased self-grooming (Fig. 1d). Our results suggest that *GABRA4* deficiency may contribute to the etiology of autism, confirming causality of *GABRA4* variations identified in the previous studies on ASD patients [33, 34]. Interestingly, *Gabra4* knockout mice showed enhanced spatial learning and memory (Fig. 1f, g). The *Gabra4* gene codes for a subunit of the GABA receptor that mediates inhibitory synaptic transmission and that contributes to tonic inhibition at extra-synaptic expression sites. Contrary to our expectation, the *Gabra4* knockout mice were resistant to the convulsive drug pentylenetetrazol (PTZ), a GABA antagonist, which causes excessive activation of the excitatory machinery (Fig. 1h). This result is consistent with previous observation that increased expression of *Gabra4* induces seizure [72]. The well-accepted hypothesis proposed to explain that seizure is an increased ratio of excitation to inhibition, which results in reduced seizure threshold. These phenotypes of *Gabra4*^*−*/*−*^ mice show HF-autism-like features: impaired social interaction and repetitive behaviors, no intellectual disability (even better than normal in spatial learning and memory) and attenuated susceptibility to seizure.

Transcriptome profiling on hippocampi of the *Gabra4*^*−/−*^ mice revealed genome-scale homeostatic regulation of gene expression, with 787 genes upregulated and 460 genes down-regulated (Fig. 2a). The upregulated genes show significant enrichments for ASD and epilepsy candidate genes and learning/memory-related genes (Fig. 2b–d), suggesting that a genome-wide homeostatic regulation of gene expression change may be involved molecular mechanism underlying the observed autistic-like behaviors in the mouse model. These upregulated genes are enriched for functions in neuronal development, which are known to be related to autism (Fig. 2e, f). Consistent with previous findings, the upregulated genes are also involved in ion transport (e.g., *Gabra2*, *Grin1*, *Kcnc1*) and synapse (e.g., *Nlgn2*, *Syp*). Many lines of evidence have suggested that synaptic dysfunction is involved in the etiology of autism [73] and intellectual disability [74]. The enrichment of human ASD and epilepsy candidate genes and LM-related genes in the hippocampal differentially expressed genes further confirms the *Gabra4*^*−*/*−*^ mouse as a potential animal model for a subtype of autism at molecular level.

A total number of 15,254 genes are expressed in the hippocampus. We mapped these genes onto the mouse interactome (obtained from BioGRID) and extracted mouse hippocampal interactome (Additional file 1: Figure S5). To illustrate the molecular mechanisms underlying the phenotypes of the mouse model, we extracted four the DEG subnetwork from the hippocampal interactome (Fig. 3a). The DEG subnetwork shows significant additional enrichments with ASD candidate genes, epilepsy candidate genes, and LM-related genes compared to all DEGs (Fig. 3b–d), suggesting that these genes function together and contribute to the etiology of this mouse model. Looking into the DEG subnetwork, we found that some well-known ASD candidate genes, such as *Kcnma1*, *Shank2*, *Cacna1a* and *Cacna1b*, and epilepsy candidate genes, such as *Scn3a*, *Grin2a*, *Gabrg2*, and *Grin2b*, are hub genes in this subnetwork. These results suggest that abnormal expression of these genes may affect signaling pathways underlying the three phenotypic domains of high-functioning autism, including ASD-like behaviors, enhanced learning/memory, and anti-epilepsy phenotype.

Besides the core features of autism, autistic patients always show a spectrum of cognitive dysfunctions and sometimes epilepsy/anti-epilepsy features. To further search for converged pathways involved in autistic-like behaviors, abnormal learning/memory and anti-epilepsy phenotypes of *Gabra4* knockout mice, we mapped autism candidate genes onto the hippocampal interactome and extracted ASD subnetwork (Fig. 4a, b), EP subnetwork (Fig. 4c, d), and LM subnetwork (Fig. 4e, f). The ASD subnetwork has interconnected many signaling pathways which are reported to be involved in autism, such as long-term potential signaling [47], glutamatergic synapses [62], and Wnt signaling [63, 64] (Fig. 5a, Additional file 2: Table S13). The EP subnetwork is enriched for pathways, such as glutamatergic [75] and dopaminergic synapse [76] pathways, which are well known to be involved in epilepsy (Fig. 5b, Additional file 2: Table S14). Among the upregulated genes, *Grin1* has the biggest betweenness values in all the subnetworks (Fig. 4a–f), suggesting the central position of *Grin1* in these subnetworks. These subnetworks have 17 nodes in common, most of which interconnect with each other to form a converged network module (Fig. 4h), with *Grin1* and *Grin2b* at the center position. The upregulation of *Grin1* and its five interactors in this converged network module may play a key role in regulating the three subnetworks underlying the three phenotypic domains. Consistent with the upregulation of NMDAR system, we found an increased NMDA/AMPA conductance ratio in KO mice (Fig. 4j). These three subnetworks share 37 enriched pathways, most of which involve NMDAR system (Fig. 5d). We also performed gene-pathway bipartite network analyses using data from KEGG database and extracted subnetworks for ASD candidate genes, EP candidate genes, and LM-related genes and the enriched pathways in these three bipartite subnetworks are largely overlapping with those in PPI subnetworks (Additional file 1: Figure S8A-H). The shared network module of these three gene-pathway bipartite subnetworks contains NMDAR-involved pathways as hubs (Additional file 1: Figure S8I), confirming that NMDAR system may play central role in regulating the pathways involved in determining the three phenotypic domains.

## Limitations

There are several limitations in this study. First, the phenotypes of the knockout mice may not accurately represent the phenotypes of autism. Three-chamber test was used to evaluate the sociability and social novelty, but the impairment of verbal communication in human autism, one of the core features of autism, was not able to be measured. Second, the Morris water maze was used to test the special learning and memory, but human intelligence was much more complicated than spatial learning and memory alone. Therefore, the results from our study on this “high-functioning autism-like” mouse model may not fully apply in the case of human autism. Third, we identified distinct and shared pathways under the three phenotypic domains of high functioning autism: autistic-like behaviors, enhanced learning/memory, and anti-epilepsy phenotype. However, such analyses were based on protein-protein interaction data and gene expression profiling. Therefore, a more detailed molecular signal transduction processes are needed in order for us to have a clear picture of the mechanism.

## Conclusion

We have shown that *Gabra4* knockout mice exhibit autistic-like behaviors and attenuated PTZ-induced seizure and enhanced learning/memory. The transcriptome sequencing on the hippocampus revealed a landscape of dysregulated genes with significant enrichment of ASD and epilepsy candidate genes. By the generation of hippocampal interactome, we have constructed subnetworks for autism candidate genes, epilepsy candidate genes, and learning/memory-related genes. These three subnetworks have a converged module with NMDAR system at central position and also share some enriched pathways involving NMDARs and their regulators, suggesting that these converged NMDAR pathways might be the commonly affected in autism spectrum disorder.

## Supplementary information

**Additional file 1: Figure S1.** Genotyping of *Gabra4*^*-/-*^ mice and behavior tests. A Mutants were identified by Sanger sequencing. B *Gabra4*^*-/-*^ mice showed no significant preference for the both chambers on the left and right (*n* = 16 for WT, and *n* = 8 for *Gabra4*^*-/-*^). No significance, Student’s *t* test. C *Gabra4*^*-/-*^ mice (*n* = 11) and wild type mice (*n* = 11) traveled similar total distance during 30-minute open field test. D During the first 5 minutes in open field test, *Gabra4*^*-/-*^ mice spent less time in the center zone compared to wild type mice. No significance, *n* = 19 for WT, *n* = 11 for *Gabra4*^*-/-*^, Student’s *t* test. E Both WT and *Gabra4*^*-/-*^ buried similar number of marbles. No significance, *n* = 10 for WT, *n* = 9 for *Gabra4*^*-/-*^ mice, Student’s *t* test. F-G *Gabra4*^*-/-*^ mice showed similar percentage of freezing time during training (F) and test day (G) as WT mice. No significance, *n* = 9 for WT, *n* = 7 for *Gabra4*^*-/-*^, Student’s *t* test. H *Gabra4*^*-/-*^ and WT displayed the similar velocity. No significance, *n* = 13 for WT, *n* = 16 for *Gabra4*^*-/-*^, Student’s *t* test. All data presented as mean ± SEM. **Figure S2.** Increased excitatory synapses. A The representative pictures of spine density of the hippocampus from wild type mice and *Gabra4*^*-/-*^ mice. Brain tissues from mice 10 weeks old were used for Golgi-Cox staining and dendritic spines were examined in Ix71 inverted microscope with a 100× objective oil immersion lens (Olympus Life Science). The number of spines per 30 μm of dendrite was compared between genotypes. B Quantification of dendritic spine density in WT versus *Gabra4*^*-/-*^ neurons. Data are presented as scatter grams (with mean ± SEM superimposed), each point corresponds to the mean spine density for a single neuron. *Gabra4*^*-/-*^ mice showed increased spine density (WT, *n* = 39 neurons from 3 animals, *Gabra4*^*-/-*^, *n* = 50 neurons from 4 animals, *p =* 0.0403, Student’s *t* test). C The representative electron micrograph shows the postsynaptic densities of the hippocampus from wild type mice and *Gabra4*^*-/-*^ mice. Scale bar, 200 nm. D Cumulative frequency distribution of PSD length in postsynaptic density of WT and *Gabra4*^*-/-*^ mice, respectively. No significant difference between WT and mutant mice (*n* = 58 for WT, and *n* = 48 for *Gabra4*^*-/-*^ mice, Student’s *t* test). E Cumulative frequency distribution of PSD thickness in postsynaptic density of WT and *Gabra4*^*-/-*^ mice, respectively. No significant difference between WT and mutant mice (*n* = 59 for WT, and *n* = 48 for *Gabra4*^*-/-*^ mice, Student’s *t* test). **Figure S3.** PCA analysis of RNAseq data, clustering of DEGs and expression level of GABA receptors and glutamate receptors. A PCA analysis of RNAseq data was performed on genes with more than 1 raw counts in at least 4 samples using plotPCA DEseq2. B Heatmap of DEGs was plotted on log2-transformed expression data using pheatmap1.0.10 package. C-E Expression levels of GABA receptors (C) and glutamate receptors (D and E) were represented by the FPKM values calculated from the RNAseq data. The *p* Values were calculated using Student's *t*-test. F-H Real-time PCRs were carried out on the differentially expressed GABA receptor *Gabra2* (F) and glutamate receptors *Grin1* (G) and *Grin2c* (H). All data presented as mean ± SEM; *n* = 7 for WT and *n* = 5 for *Gabra4*^*-/-*^ mice; The *p* Values were calculated using Student's *t*-test. **Figure S4.** Distinct functions between upregulated and downregulated genes (DEGs). A Schizophrenia candidate genes are enriched in *Gabra4*^*-/*-^ differential expression genes. Error bars represent the standard error of the fraction, estimated using bootstrapping method with 100 resamplings. **p < 0.01, Fisher’s exact test. B The enriched cellular components of GO terms with DEGs. The top 10 enriched terms with up- and down-regulated genes. C The enriched molecular functions of GO terms with DEGs. The top 10 enriched terms with up- and down-regulated genes. **Figure S5.** Construction of hippocampal interactome network and extraction of DEG subnetwork, ASD subnetwork, LM subnetwork and EP subnetwork. The mouse hippocampal interactome, which contained 4,202 nodes and 9,205 edges, was constructed by integrating the hippocampal expressed genes and a protein interaction data from BioGRID (Biological General Repository for Interaction Datasets). Four subnetworks were extracted by mapping the “seeds” (DEGs, ASD candidate genes, LM-related genes or EP candidate genes) on to the hippocampal interactome network to include the interconnected “seeds” and their co-expressed neighbors in the hippocampal interactome network. **Figure S6.** Subnetworks extracted from randomized PPI networks compared to real subnetworks. Mouse PPI interactome from BioGRID was randomized for 1,000 times by shuffling nodes and 4 sets of seeds (DEGs, ASD candidates, EP candidates, LM candidates) were mapped onto the 1,000 randomized networks to extract subnetworks as controls. The size distribution of each control set of subnetworks was plotted in comparison with the size of each real subnetwork. Blue lines indicate the mean sizes of the control subnetworks, and red arrows indicate the sizes of the real subnetworks. A Node counts distribution of DEG subnetworks extracted from randomized networks. B Edge counts distribution of DEG subnetworks extracted from randomized networks. C Node counts distribution of ASD subnetworks extracted from randomized networks. D Edge counts distribution of ASD subnetworks extracted from randomized networks. E Node counts distribution of EP subnetworks extracted from randomized networks. F Edge counts distribution of EP subnetworks extracted from randomized networks. G Node counts distribution of LM subnetworks extracted from randomized networks. H Edge counts distribution of LM subnetworks extracted from randomized networks. **Figure S7.** Comparison of the enriched pathways with ASD, epilepsy and LM subnetworks. The comparison of enriched KEGG pathways with the three subnetworks. There are 37 enriched pathways shared by three subnetworks. The enrichment analysis was performed using DAVID bioinformatics tool with a *p*-value cutoff of 0.05 and FDR less than 0.05. ASD: autism; EP: epilepsy; LM: learning/memory. **Figure S8.** Gene-pathway bipartite networks for DEGs, ASD candidates, EP candidates, LM-related genes. A DEG-pathway bipartite subnetwork. DEGs were mapped onto the hippocampal expressed gene-pathway bipartite network to extract a subnetwork including DEGs and their involved pathways, and the pathways significantly enriched with DEGs (*p* < 10^-6^) and containing at least 10 seed genes were kept. B Comparison of enriched pathways in DEGs PPI subnetwork and DEG-pathway bipartite subnetwork. C ASD gene-pathway bipartite subnetwork. ASD candidate genes were mapped onto the hippocampal expressed gene-pathway bipartite network to extract a subnetwork including ASD candidate genes and their involved pathways, and the pathways significantly enriched with ASD candidate genes (*p* < 10^-6^) and containing at least 10 seed genes were kept. D Comparison of pathways enriched in ASD PPI subnetwork and ASD gene-pathway bipartite subnetwork. E EP gene-pathway bipartite subnetwork. EP candidate genes were mapped onto the hippocampal expressed gene-pathway bipartite network to extract a subnetwork including EP candidate genes and their involved pathways, and the pathways significantly enriched with EP candidate genes (*p* < 10^-6^) and containing at least 10 seed genes were kept. F Comparison of pathways involved in EP PPI subnetwork and EP gene-pathway bipartite subnetwork. G LM gene-pathway bipartite subnetwork. LM-related genes were mapped onto the hippocampal expressed gene-pathway bipartite network to extract a subnetwork including LM-related genes and their involved pathways, and the pathways significantly enriched with LM-related genes (*p* < 10^-6^) and containing at least 10 seed genes were kept. H Comparison of pathways involved in LM PPI subnetwork and LM gene-pathway bipartite subnetwork. I The network module shared by all three gene-pathway bipartite subnetworks. Fisher’s exact test was used for calculating all the *p* values.

**Additional file 2: Table S1.** The 15,254 expressed genes. **Table S2.** The gene list of hippocampal interactome. **Table S3.** The 9205 interactions among the expressed genes. **Table S4.** The 1247 differentially expressed genes. **Table S5.** The 787 upregulated genes. **Table S6.** The 460 downregulated genes. **Table S7.** The gene list of DEG subnetwork. **Table S8.** The gene list of ASD subnetwork. **Table S9.** The list of 647 epilepsy candidate genes. **Table S10.** The gene list of epilepsy subnetwork. **Table S11.** The list of 909 learning/memory related genes. **Table S12.** The gene list of learning/memory subnetwork. **Table S13.** The KEGG clusters involved in ASD subnetwork. **Table S14.** The KEGG clusters involved in epilepsy subnetwork. **Table S15.** The KEGG clusters involved in learning/memory subnetwork. **Table S16.** The common KEGG clusters involved in three subnetworks. **Table S17.** Hippocampal gene-pathway bipartite network. **Table S18.** Hippocampal DEG gene-pathway bipartite network. **Table S19.** Hippocampal ASD gene-pathway bipartite network. **Table S20.** Hippocampal EP gene-pathway bipartite network. **Table S21.** Hippocampal LM gene-pathway bipartite network. **Table S22.** Genes involved in DEG gene-pathway bipartite network. **Table S23.** Genes involved in ASD gene-pathway bipartite network. **Table S24.** Genes involved in EP gene-pathway bipartite network. **Table S25.** Genes involved in LM gene-pathway bipartite network. **Table S26.** Hippocampal converged-pathway bipartite network module. **Table S27.** Genes involved in converged gene-pathway bipartite network module. **Table S28.** Comparison of enriched pathways in DEG-pathway bipartite network and those enriched in DEGs PPI network. **Table S29.** Comparison of enriched pathways in ASD gene-pathway bipartite network and those enriched in ASD PPI network. **Table S30.** Comparison of enriched pathways in EP gene-pathway bipartite network and those enriched in EP PPI network. **Table S31.** Comparison of enriched pathways in LM gene-pathway bipartite network and those enriched in LM PPI network.

## Data Availability

The datasets used and/or analyzed during the current study are available from the corresponding author on reasonable request.

## Abbreviations

AMPAR: α-Amino-3-hydroxy-5-methyl-4-isoxazole-propionic acid receptor
ANOVA: Analysis of variance
ASD: Autism spectrum disorder
BioGRID: Biological General Repository for Interaction Datasets
cAMP: Cyclic adenosine monophosphate
CGH: Comparative genomic hybridization
CNV: Copy number variations
DAVID: Database for Annotation, Visualization and Integrated Discovery
DEG: Differentially expressed gene
DG: Dentate gyrus
EP: Epilepsy
EPM: Elevated plus maze
FDR: False discovery rate
FPKM: Fragments per kilobase of transcript per million mapped reads
GABA: γ-Aminobutyric acid
GO: Gene ontology
HF-ASD: High-functioning autism
IQ: Intelligence quotient
KEGG: Kyoto Encyclopedia of Genes and Genomes
LM: Learning/memory
MAPK: Mitogen-activated protein kinase
NMDAR: *N*-methyl-d-aspartate receptor
OFT: Open field test
PPI: Protein-protein interaction
PTZ: Pentylenetetrazol
TALEN: Transcription activator-like (TAL) effector nucleases
WES: Whole exome sequencing
WGCNA: Weighted correlation network analysis
WT: Wild type
