# Supplementary material for: Transcriptomics of Gabra4 knockout mice reveals common NMDAR pathways underlying autism, memory, and epilepsy
Source: Mol Autism. 2020 Feb 7;11:13. doi: 10.1186/s13229-020-0318-9 (PMC7007694; doi:10.1186/s13229-020-0318-9)
Supplement: Supplementary file 1 — Additional file 1: Figure S1. Genotyping of Gabra4-/- mice and behavior tests. A Mutants were identified by Sanger sequencing. B Gabra4-/- mice showed no significant preference for the both chambers on the left and right (n = 16 for WT, and n = 8 for Gabra4-/-). No significance, Student’s t test. C Gabra4-/- mice (n = 11) and wild type mice (n = 11) traveled similar total distance during 30-minute open field test. D During the first 5 minutes in open field test, Gabra4-/- mice spent less time in the center zone compared to wild type mice. No significance, n = 19 for WT, n = 11 for Gabra4-/-, Student’s t test. E Both WT and Gabra4-/- buried similar number of marbles. No significance, n = 10 for WT, n = 9 for Gabra4-/- mice, Student’s t test. F-G Gabra4-/- mice showed similar percentage of freezing time during training (F) and test day (G) as WT mice. No significance, n = 9 for WT, n = 7 for Gabra4-/-, Student’s t test. H Gabra4-/- and WT displayed the similar velocity. No significance, n = 13 for WT, n = 16 for Gabra4-/-, Student’s t test. All data presented as mean ± SEM. Figure S2. Increased excitatory synapses. A The representative pictures of spine density of the hippocampus from wild type mice and Gabra4-/- mice. Brain tissues from mice 10 weeks old were used for Golgi-Cox staining and dendritic spines were examined in Ix71 inverted microscope with a 100× objective oil immersion lens (Olympus Life Science). The number of spines per 30 μm of dendrite was compared between genotypes. B Quantification of dendritic spine density in WT versus Gabra4-/- neurons. Data are presented as scatter grams (with mean ± SEM superimposed), each point corresponds to the mean spine density for a single neuron. Gabra4-/- mice showed increased spine density (WT, n = 39 neurons from 3 animals, Gabra4-/-, n = 50 neurons from 4 animals, p = 0.0403, Student’s t test). C The representative electron micrograph shows the postsynaptic densities of the hippocampus from wild type mice and Gabr [file 13229_2020_318_MOESM1_ESM.docx]

***Additional File 1***

**Title: Transcriptomics of *Gabra4* knockout mice reveals common NMDAR pathways underlying autism, memory and epilepsy**

**Authors:**

Cuixia Fan^1,2,#^, Yue Gao^1,3,4,#^, Guanmei Liang^1,3,4^, Lang Huang^3,5^, Jing Wang^1^, Xiaoxue Yang^1^, Yiwu Shi^2^, Ursula C. Dräger^6^, Mei Zhong^1^, Tian-Ming Gao^3,5^ and Xinping Yang^1,3,4,*^

**Affiliations**

^1^Department of Obstetrics and Gynecology, Nanfang Hospital, Southern Medical University, Guangzhou 510515, China

^2^Institute of Neuroscience and Department of Neurology, The Second Affiliated Hospital of Guangzhou Medical University, Guangzhou, 510260, China

^3^Key Laboratory of Mental Health of the Ministry of Education, Southern Medical University, Guangzhou 510515, China

^4^Department of Bioinformatics, School of Basic Medical Sciences, Southern Medical University, Guangzhou 510515, China

^5^State Key Laboratory of Organ Failure Research, Guangdong-Hong Kong-Macao Greater Bay Area Center for Brain Science and Brain-Inspired Intelligence, Guangdong Key Laboratory of Psychiatric Disorders, Collaborative Innovation Center for Brain Science, Department of Neurobiology, School of Basic Medical Sciences, Southern Medical University, Guangzhou 510515, China

^6^Department of Psychiatry, University of Massachusetts Medical School, Worcester MA 01655, USA

^#^ These authors contributed equally.

^*^Corresponding author, Xinping Yang, Southern Medical University, 1838 N. Guangzhou Ave, Guangzhou 510515, China; Tel, (86) 20-6278-6524; Email: xp[yang1@smu.edu.cn](mailto:yangxinping@smu.edu.cn).

**TABLE OF CONTENTS**

**Methods**

1. Generation of *gabra4* knockout mice

2. Three-chamber test

3. Self-grooming test

4. Marble burying test

5. Open field test

6. Elevated plus test

7. Y maze spontaneous alternation test

8. Fear conditioning test

9. Morris water maze

10. RNA preparation

11. RNA-seq and differential expression analysis

12.Functional analysis of differential expressed genes

13. Enrichment for candidate genes of ASD, epilepsy and schizophrenia

14. Construction of hippocampal interactome and co-expression network

15. Construction of DEG subnetwork, ASD subnetwork, EP subnetwork and LM subnetwork

16. Data sources for disease candidate genes

17. Slice preparation and electrophysiology

18. The primers for qRT-PCR

**Figures**

Additional file 1: Figure S1 Genotyping of Gabra4^-/-^ mice and behavior tests.

Additional file 1: Figure S2 Increased excitatory synapses.

Additional file 1: Figure S3 PCA analysis of RNAseq data, clustering of DEGs and expression level of GABA receptors and glutamate receptors.

Additional file 1: Figure S4 Distinct functions between upregulated and downregulated genes (DEGs).

Additional file 1: Figure S5 Construction of hippocampal interactome network and extraction of DEG subnetwork, ASD subnetwork, LM subnetwork and EP subnetwork.

Additional file 1: Subnetworks extracted from randomized PPI networks compared to real subnetworks.

Additional file 1: Figure S7 Comparison of the enriched pathways with ASD, epilepsy and LM subnetworks.

Additional file 1: Figure S8 Gene-pathway bipartite networks for DEGs, ASD candidates, EP candidates, LM-related genes.

**Tables**

Additional file 2: Table S1 The 15,254 expressed genes.

Additional file 2: Table S2 The gene list of hippocampal interactome.

Additional file 2: Table S3 The 9,205 interactions among the expressed genes.

Additional file 2: Table S4 The 1,247 differentially expressed genes.

Additional file 2: Table S5 The 787 upregulated genes.

Additional file 2: Table S6 The 460 downregulated genes.

Additional file 2: Table S7 The gene list of DEG subnetwork.

Additional file 2: Table S8 The gene list of ASD subnetwork.

Additional file 2: Table S9 The list of 647 epilepsy candidate genes.

Additional file 2: Table S10 The gene list of epilepsy subnetwork.

Additional file 2: Table S11 The list of 909 learning/memory related genes.

Additional file 2: Table S12 The gene list of learning/memory subnetwork.

Additional file 2: Table S13 The KEGG clusters involved in ASD subnetwork.

Additional file 2: Table S14 The KEGG clusters involved in epilepsy subnetwork.

Additional file 2: Table S15 The KEGG clusters involved in learning/memory subnetwork.

Additional file 2: Table S16 The common KEGG clusters involved in three subnetworks.

Additional file 2: Table S17: Hippocampal gene-pathway bipartite network.

Additional file 2: Table S18 Hippocampal DEG gene-pathway bipartite network.

Additional file 2: Table S19 Hippocampal ASD gene-pathway bipartite network.

Additional file 2: Table S20 Hippocampal EP gene-pathway bipartite network.

Additional file 2: Table S21 Hippocampal LM gene-pathway bipartite network.

Additional file 2: Table 22: Genes involved in DEG gene-pathway bipartite network.

Additional file 2: Table 23: Genes involved in ASD gene-pathway bipartite network.

Additional file 2: Table 24: Genes involved in EP gene-pathway bipartite network.

Additional file 2: Table 25: Genes involved in LM gene-pathway bipartite network.

Additional file 2: Table 26: Hippocampal converged-pathway bipartite network module.

Additional file 2: Table 27: Genes involved in converged gene-pathway bipartite network module.

Additional file 2: Table S28: Comparison of enriched pathways in DEG-pathway bipartite network and those enriched in DEGs PPI network.

Additional file 2: Table S29: Comparison of enriched pathways in ASD gene-pathway bipartite network and those enriched in ASD PPI network.

Additional file 2: Table S30: Comparison of enriched pathways in EP gene-pathway bipartite network and those enriched in EP PPI network.

Additional file 2: Table S31: Comparison of enriched pathways in LM gene-pathway bipartite network and those enriched in LM PPI network.

**References**

**Methods**

**1. Generation of *Gabra4* knockout mice**

The generation of *Gabra4* knockout C57BL/6 mice by TALEN technology [[1](#_ENREF_1)] was carried out in Cyagen Biosciences Inc.(China). Briefly, exon 1 of mouse *Gabra4* gene (GenBank accession number: NM_010251.2) was selected as target site, and TALEN mRNAs generated by in vitro transcription were then injected into fertilized eggs for KO mouse productions (**S1A Fig**). The PCR was used to amplify the DNA fragment containing the target site for the identification of *Gabra4^-/-^* mice using forward primer: 5’-CGAGAGGCTGGAAACGTGAACA-3’and reverse primer:5’-CTGAGTCTACTCGGTCAAAGGAAAGC-3’. The PCR products were Sanger-sequenced to confirm the deletion.

**2. Three-Chamber test**

Mice were tested for sociability as described previously [[2](#_ENREF_2)]. Specifically, the testing apparatus was a rectangular clear Plexiglas box containing three chambers (60cm (L) x 40cm (W) x 20 cm (H)). The dividing walls had doorways allowing the mouse access to each chamber. Age and sex matched animals were used for all tests. Mice from the same strain were used as stranger mice and were habituated to being placed inside the wire cage for 5 days prior to beginning of testing. Each test mouse was first placed into the center chamber with open access to both left and right chambers, each chamber containing an empty round wire cage. The wire cage (12cm (H), 11cm diameter) allows nose contact between mice but prevents fighting. After 10 min of habituation, during the social phase, an age-matched stranger was placed into one wire cage, while the opposite remained empty. The test mouse was allowed to freely explore the social apparatus for 10 min and to show whether it preferred to interact with the object or with the stranger mouse. At the end of the first 10 minutes, each mouse was tested in a second 10-min session to evaluate the preference for a novel stranger, which was then placed into the opposite wire cage. The times spent in sniffing at different test chambers were measured.

**3. Self-grooming test**

This test was performed as previously described [[3](#_ENREF_3)]. Each subject was placed individually into a clean standard cage and allowed to get used to it for 10 min. Following this habituation period, the subjects were observed for another 10 min, during which the cumulative times spent in self-grooming were scored by an experimenter sitting approximately 2 meters from the test cage. The test sessions were videotaped.

**4. Marble burying test**

Marble burying is was conducted as previously described [[4](#_ENREF_4)]. Mice were introduced individually in transparent cages (40×30×22 cm) containing 20 black glass marbles (diameter, 1.4 cm) equidistant in a 4×5 arrangement on 5cm deep fresh sawdust. To prevent escape, each cage was covered with a filtering lid. The animals were removed from the cages after 30 min, and the cages with more than 2/3 of the marbles buried in sawdust were taken as positive test results.

**5. Open field test**

This test was conducted as previously described [[5](#_ENREF_5)]. Animal activity was recorded using the VersaMax Animal Activity Monitoring System (AccuScan Instruments, Columbus, OH, USA), which included an empty clear Plexiglas (41×41×30 cm) open-field arena. Each subject was placed in the box for 30 minutes. We analyzed total distance, and time spent and distance moved during the first 5 minutes in central zones of the field.

**6. Elevated plus maze test**

Elevated plus maze testing was performed as described [[6](#_ENREF_6)]. Brieﬂy, mice were placed in the junction of the elevated plus maze apparatus, facing an open arm, and mouse movements were recorded with Noldus software for a 5 min trial. Mice were determined to be on an open arm when all four limbs were fully on the open arm.

**7. Y maze spontaneous alternation Test**

Y-maze spontaneous alternation test was performed as previously described [[7](#_ENREF_7)]. The Y-shaped maze with three white, opaque plastic arms at a 120° angle from each other. The mouse for test was placed at the center of the maze and allowed to freely explore the three arms. An entry is considered to occur when all four limbs are within the arm. The number of arm entries and the number of triads are recorded in order to calculate the percentage of alternation.

**8. Fear conditioning test**

Fear conditioning was performed as previously described [[8](#_ENREF_8)]. This test was performed over 2 consecutive days using the Contextual NIR Video Fear Conditioning System for Mouse and Video Freeze Software (Med Associates). For training on day 1, subjects were placed into the Context A and left to explore for 2 min before tone onset (20 sec, 75 dB, 2800 Hz). For delay conditioning, the termination of a 30-sec tone (conditioning stimulus, CS) was contiguous with a 0.75-mA, 2-sec foot shock (the unconditioning stimulus, US). This CS-US pairing was repeated 3 times. The delay conditioning took place at 60-secinter-trial intervals, defined as the time between CS onset and the next CS onset. After the last shock, animals were left for 60 sec and then returned to their home cages. For testing on Day 2 (24 hours after training), the animals were placed in a separate room into 4 novel and structurally distinct chambers (Context B). After 120 sec in Context B, each mouse was exposed to 180-sec tone stimuli, but with the shock omitted. The freezing rate of mice was recorded.

**9. Morris water maze**

Spatial learning was conducted as previously described [[9](#_ENREF_9)]. Briefly, a test mouse was trained to find a hidden circular platform (10 cm in diameter) in a 120cm-diameter circular pool filled with white opaque water (22℃±1). The testing room was filled with a number of extra maze cues. Training was performed over 6 consecutive days with 4 trails per day. Subjects were pseudo-randomly placed into each of four starting locations of platform (SE, NE, SW, NW) for daily training trials. For each trial, mice were remained on the hidden circular platform for 15sec until they found the hidden platform or were guided to it by the experimenter if not found within 60sec. The subject rest at least 30sec before a new trail starting. All data was record by the Water Maze video tracking software (Digbehv, Jlecgonline, Shanghai). Daily data of the averaged across the four trials was calculated. The time (s) and speed (mm/s) of escape latency to find the platform were automatically measured for each trail. A probe trial was assessed on day 7; first, mice were placed into the quadrant opposite of the hidden platform with a “flag” mounted to test the swimming speed, and then removed the hidden platform, mice were placed in the pool and allowed to swim for 60sec, the numbers of crossing over the trained platform position and percent time of each quadrants were automatically recorded.

**10. RNA preparation**

For each genotype, three RNA samples were prepared. Each RNA sample was extracted from dissected hippocampi of 3 adult mice according to the manufacturer’s protocol (RNA easy Mini Kit, Qiagen, USA). The quality and yield of the isolated RNAs was assessed using a NanoDrop Spectrophotometer (Thermo Fisher Scientific, Waltham, MA, USA) and Agilent 2100 Bioanalyzer (Agilent Technologies, Santa Clara, CA, USA). Only RNAs with a high RNA integrity number (RIN>9) were selected and used for the subsequent sequencing.

**11. RNA-seq and differential expression analysis**

Sequencing libraries were generated using NEB Next Ultra^TM^ RNA Library Prep Kit for Illumima (NEB, USA) following manufacturer’s recommendations and index codes were added to attribute sequences to each sample. For each sample, 3 µg RNA was used as input material for library preparation. RNA sequencing was performed at Berry Genomics (Beijing, China) using Illumina NovaSeq. Paired-end sequences were obtained at a read length of 150bp with 30,569,087 mean read pairs (a range from 22,827,629 to 37,525,418) per sample. Raw data (raw reads) of fastq format were firstly processed through in-house Perl scripts to remove reads containing adapters, reads containing poly-N and reads with low quality. At the same time, Q20, Q30 and GC content the clean data were calculated using FastQC (v0.11.5) and duplicate reads were marked by Picard (v2.18.6). All the downstream analyses were based on the clean data with high quality. Reference genome (mm10) and gene model annotation files were downloaded from UCSC Genome Browser (http://hgdownload.soe.ucsc.edu/downloads.html#mouse). Index of the reference genome was built using STAR (v2.5.2) and paired-end clean reads were aligned to the reference genome using STAR (v2.5.2) [[10](#_ENREF_10)]. Reads numbers mapped to each gene were counted using HTseq-count (v0.9.0) (https://htseq.readthedocs.io/en/release_0.9.0/count.html). And then FPKM of each gene was calculated using counts2fpkm function (Tmisc v0.1.19) (https://www.rdocumentation.org/packages/Tmisc/versions/0.1.19). Differential expression analysis on two groups was performed using the DESeq2 (v1.20.0) (https://www.rdocumentation.org/packages/DESeq/versions/1.24.0) and the edgeR (v3.22.5) (https://anaconda.org/bioconda/bioconductor-edger). Differentially expressed genes (DEGs) were determined using a cutoff of adjusted *p*<0.05 for DESeq2 and *p*<0.01 for edgeR.

**12. Functional analysis of differentially expressed genes**

Functional annotations of up-regulated and down-regulated genes were done using Database for Annotation, Visualization and Integrated Discovery (DAVID) tools (v6.8) and terms were identified with FDR (False Discovery Rate) less than 0.05. Visualization and plot of top selected terms were done using ggplot2 package (v3.0.0).

**13. Enrichment for candidate genes of ASD, epilepsy and schizophrenia**

The enrichment of disease-related genes in DEGs was calculated as describe in following steps, (1) the expected disease candidate genes is disease candidates in expressed genes; (2) the observed disease candidate genes is the candidate genes in DEGs; (3) *p*-value was calculated using Fisher’s exact test. The same method was used for up-regulated genes and down-regulated genes.

**14. Construction of hippocampal interactome and co-expression network**

We constructed a hippocampal interactome by mapping 15,254 expressed genes (**Additional file 2: Table S1**) from the mouse hippocampal transcriptome to the whole mouse interactome from BioGRID (Biological General Repository for Interaction Datasets, <http://thebiogrid.org>), which contains 4,204 nodes (**Additional file 2: Table S2**) and 9,205 edges (**Additional file 2: Table S3**). We calculated correlation coefficient using FPKM value (fragments per kilobase of transcript per million mapped reads) and constructed a co-expression network using a cutoff 0.75 of correlation coefficient using WGCNA (<https://horvath.genetics.ucla.edu/html/CoexpressionNetwork/Rpackages/WGCNA/>) (**Additional file 2: Table S5**).

**15. Construction of DEG subnetwork, ASD subnetwork, EP subnetwork and LM subnetwork**

To construct a subnetwork for differentially expressed genes (DEG subnetwork), we mapped the 1,247 DEGs to the mouse hippocampal interactome to extract hippocampal DEG interactome network containing these DEGs and their first neighbors if they have both physical interaction and co-expression relationship. For extraction of the networks, the self-loop edges and zero-degree nodes were removed. To construct a subnetwork for ASD candidate genes (ASD subnetwork), we mapped the 1,036 ASD candidate genes to the mouse hippocampal interactome to extract a subnetwork containing these candidate genes and their first neighbors if they have both physical interaction and co-expression relationship. The same method was also applied for extracting subnetwork for epilepsy (EP subnetwork) and subnetwork for learning/memory (LM subnetwork) using 647 epilepsy and 909LM candidate genes as we described above.

**16. Data sources for disease candidate genes:**

1. AutDB database, <http://autism.mindspec.org/autdb/HG_Home.do>;

2. Schizophrenia Gene Resource, <https://bioinfo.uth.edu/SZGR/>;

3. Epilepsy candidate genes (**Additional file 2: Table S9**):

(1) HGMD (The Human Gene Mutation Database, <http://www.hgmd.cf.ac.uk/>);

(2) OMIM (Online Mendelian Inheritance in Man,[www.omim.org](http://www.omim.org));

(3) PhenGenI (Phenotype-Genotype Integrator, <https://www.ncbi.nlm.nih.gov/gap/phegeni>).Key word is ‘epilepsy’ for three databases.

4. Learning/memory (LM) related genes: We compiled a list of 909 high-functioning genes (**Additional file 2: Table S11**) by manually searching PubMed using “high functioning autism”, “Asperger syndrome”, “atypical autism”, “better/enhanced/improved learning/memory”, “better/enhanced/improved cognition” and KEGG map using “memory”, “learning”.

**17. Slice Preparation and Electrophysiology**

Mice were anesthetized deeply with isoflurane and then decapitated. The brain was quickly removed out and transferred to ice-cold oxygenated artificial cerebrospinal fluid (ASCF) containing (in mM):220 sucrose, 2.5 KCl, 1.3 CaCl_2_, 2.5 MgSO_4_, 1 NaH_2_PO_4_, 26 NaHCO_3_, and 10 glucose. The VT-1200S vibratome (Leica, Germany) was used to cut 300-μm coronal hippocampal slices. Hippocampal slices were incubated in ASCF containing (in mM): 126 NaCl, 3 KCl, 1.25 NaH_2_PO_4_, 1.0 MgSO_4_, 2.0 CaCl_2_, 26 NaHCO_3_, and 10 Glucose at 34℃ for 30 min. Then slices were put at RT (25 ± 1℃) for 2 to 8h. All extracellular solutions were constantly carbonated (95% O_2_, 5% CO_2_). Whole-cell patch-clamp recordings of hippocampus pyramidal neurons were carried out as previously described [[11](#_ENREF_11)]. During recordings, the recording chamber was continuously perfused with ASCF (2 ml/min) saturated with 95% O_2_/ 5% CO_2_ at 32-34℃. The hippocampus pyramidal neurons were recorded using a MultiClamp 700B amplifier and 1440A digitizer (Molecular Device) under IR-DIC visualization (Zeiss, Axioskop 2). The glass pipettes (resistance of 3–6 MΩ) were pulled with a micropipette puller (P-97, Sutter instrument). For evoke EPSCs, synaptic responses were evoked by a stimulating electrode placed in the DG fiber path approximately 0.2 mm away from the recorded cell bodies in the CA1. 20 μM bicuculline was used to isolate excitatory synaptic currents. The internal solution contained 115 mM cesium methanesulonate, 20 mMCsCl, 10 mM HEPES, 2.5 mM MgCl2, 10 mM sodium phosphocreatine, 5 mM QX-314, 4 mM Na2-ATP, 0.4 mM Na3GTP, and 0.6 mM EGTA (pH 7.3, 285 mOsm). AMPA receptor-mediated currents were recorded at −70 mV. NMDA receptor-mediated currents were voltage-clamped at +40 mV, and mean response size was measured between 110-160 ms after the peak current. Currents were filtered at 3 kHz with a low-pass filter, and data were digitized at 10 kHz and acquired using the pCLAMP 10 software. Series resistance was continuously monitored for each neuron. The neuron was rejected from statistical analysis if the series resistance increased more than 20%. Data analyses were conducted using the Mini Analysis software (Synaptosoft).

**18. The primers for qRT-PCR**

The list of primers for qRT-PCR.

Gene Forward Reverse

Grin1 ATGCACCTGCTGACATTCG TATTGGCCTGGTTTACTGCCT

Grin2c GGGATCTGCCATAACGAGAAG GCACTGAGTGTCGAAGTTTCCA

Gabra2 AGTCAGTGGCCCATAACATGA TTGTGTAAGCGTAGCTTCCAAA

Gapdh CGTGGAGTCTACTGGTGT TGTCATATTTCTCGTGGT

**Figures**

**
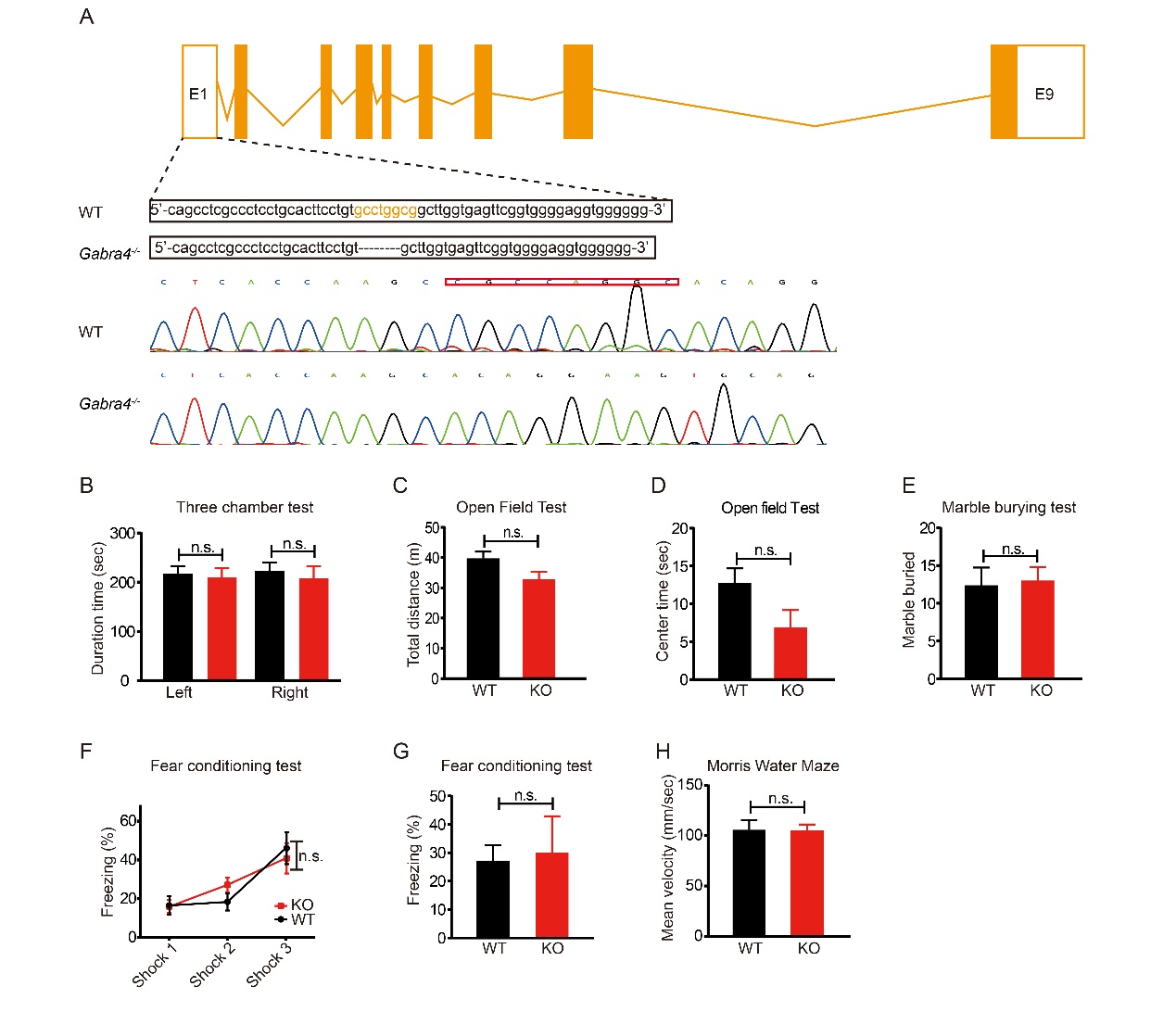
**

**Figure S1 Genotyping of *Gabra4^-/-^* mice and behavior tests.**

**A** Mutants were identified by Sanger sequencing.

**B** *Gabra4^-/-^* mice showed no significant preference for the both chambers on the left and right (*n* = 16 for WT, and *n* = 8 for *Gabra4^-/-^*). No significance, Student’s *t* test.

**C** *Gabra4^-/-^* mice (*n* = 11) and wild type mice (*n* = 11) traveled similar total distance during 30-minute open field test.

**D** During the first 5 minutes in open field test, *Gabra4^-/-^* mice spent less time in the center zone compared to wild type mice. No significance, *n* = 19 for WT, *n* = 11 for *Gabra4^-/-^*, Student’s *t* test.

**E** Both WT and *Gabra4^-/-^* buried similar number of marbles. No significance, *n* = 10 for WT, *n* = 9 for *Gabra4^-/-^* mice, Student’s *t* test.

**F-G** *Gabra4^-/-^* mice showed similar percentage of freezing time during training **(F)** and test day **(G)** as WT mice. No significance, *n* = 9 for WT, *n* = 7 for *Gabra4^-/-^*, Student’s *t* test.

**H** *Gabra4^-/-^* and WT displayed the similar velocity. No significance, *n* = 13 for WT, *n* = 16 for *Gabra4^-/-^*, Student’s *t* test.

All data presented as mean ± SEM.

**
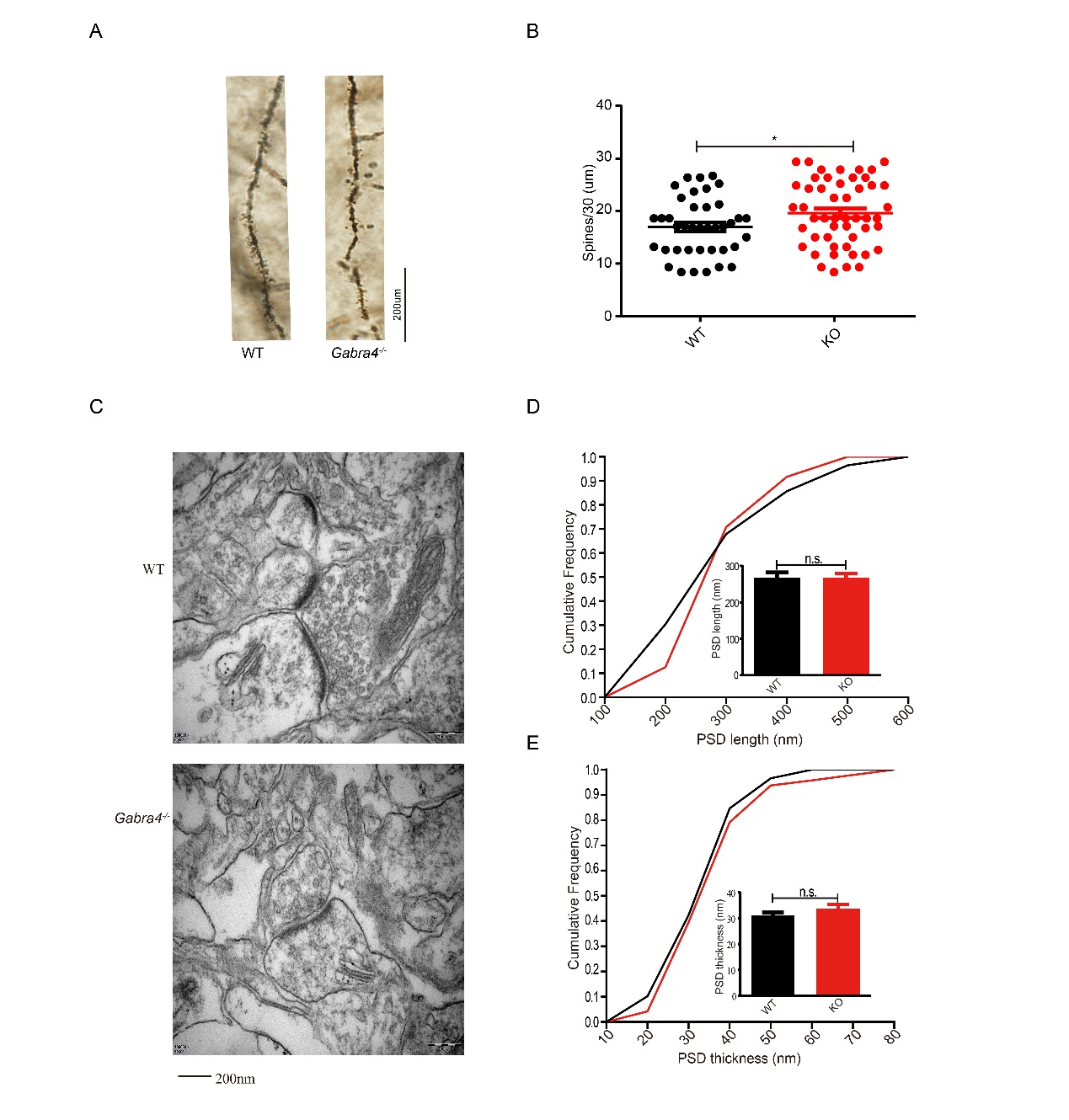
**

**Figure S2 Increased excitatory synapses.**

**A** The representative pictures of spine density of the hippocampus from wild type mice and *Gabra4^-/-^* mice. Brain tissues from mice 10 weeks old were used for Golgi-Cox staining and dendritic spines were examined in Ix71 inverted microscope with a 100× objective oil immersion lens (Olympus Life Science). The number of spines per 30 μm of dendrite was compared between genotypes.

**B** Quantification of dendritic spine density in WT versus *Gabra4^-/-^* neurons. Data are presented as scattergrams (with mean ± SEM superimposed), each point corresponds to the mean spine density for a single neuron. *Gabra4^-/-^* mice showed increased spine density (WT, *n* = 39 neurons from 3 animals, *Gabra4^-/-^*, *n* = 50 neurons from 4 animals, *p =* 0.0403, Student’s *t* test).

**C** The representative electron micrograph shows the postsynaptic densities of the hippocampus from wild type mice and *Gabra4^-/-^* mice. Scale bar, 200 nm.

**D** Cumulative frequency distribution of PSD length in postsynaptic density of WT and *Gabra4^-/-^* mice, respectively. No significant difference between WT and mutant mice (*n* = 58 for WT, and *n* = 48 for *Gabra4^-/-^* mice, Student’s *t* test).

**E** Cumulative frequency distribution of PSD thickness in postsynaptic density of WT and *Gabra4^-/-^* mice, respectively. No significant difference between WT and mutant mice (*n* = 59 for WT, and *n* = 48 for *Gabra4^-/-^* mice, Student’s *t* test).


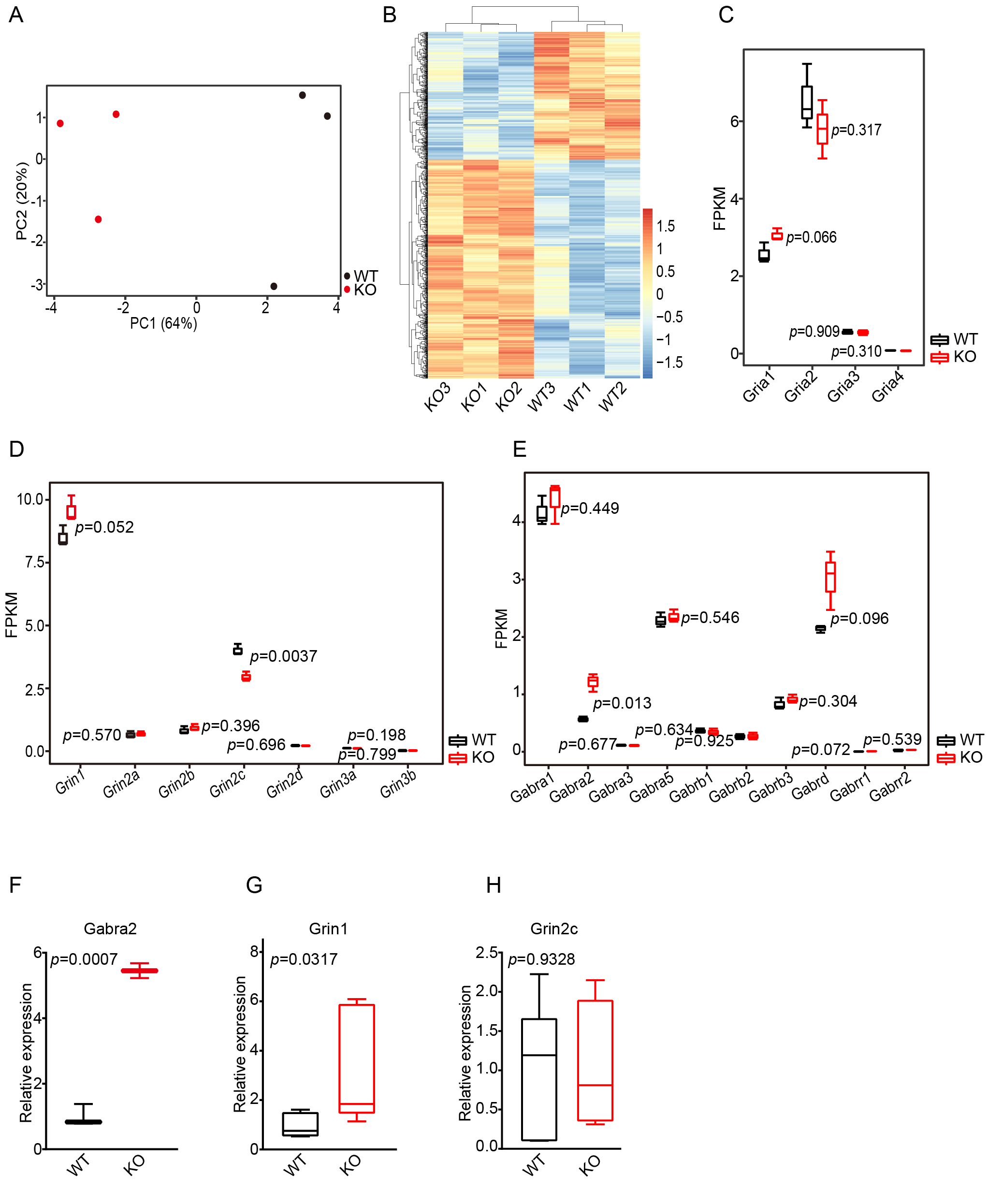


**Figure S3 PCA analysis of RNAseq data, clustering of DEGs and expression level of GABA receptors and glutamate receptors.**

**A** PCA analysis of RNAseq data was performed on genes with more than 1 raw counts in at least 4 samples using plotPCA DEseq2.

**B** Heatmap of DEGs was plotted on log2-transformed expression data using pheatmap1.0.10 package.

**C-E** Expression levels of GABA receptors (**C**) and glutamate receptors (**D** and **E**) were represented by the FPKM values calculated from the RNAseq data. The *p* Values were calculated using Student's *t*-test.

**F-H** Real-time PCRs were carried out on the differentially expressed GABA receptor *Gabra2* (**F**) and glutamate receptors *Grin1* (**G**) and *Grin2c* (**H**). All data presented as mean ± SEM; *n* = 7 for WT and *n* = 5 for *Gabra4^-/-^* mice; The *p* Values were calculated using Student's *t*-test.

**
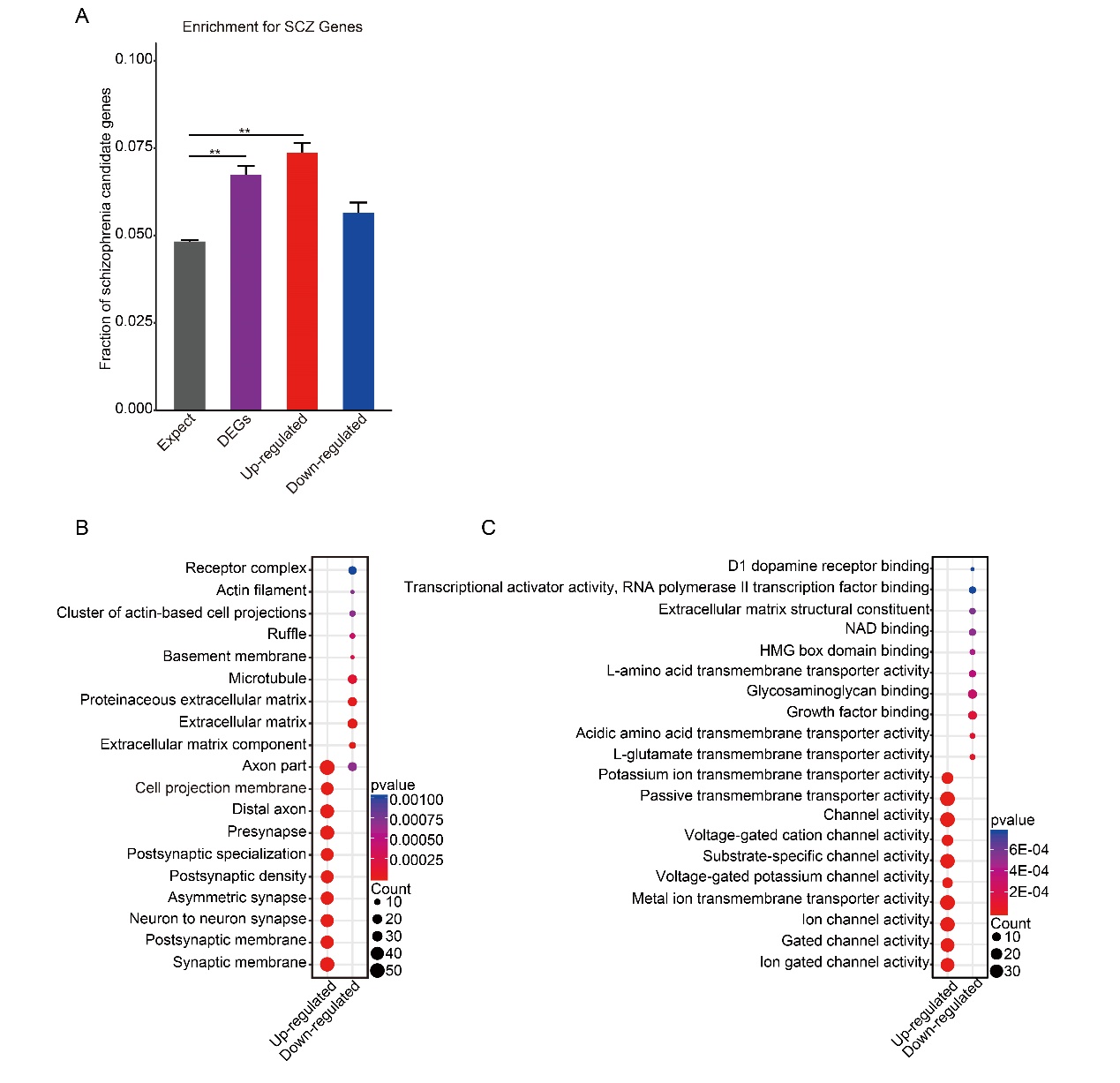
**

**Figure S4 Distinct functions between upregulated and downregulated genes (DEGs).**

**A** Schizophrenia candidate genes are enriched in *Gabra4^-/^*^-^ differential expression genes. Error bars represent the standard error of the fraction, estimated using bootstrapping method with 100 resamplings. ***p <* 0.01, Fisher’s exact test.

**B** The enriched cellular components of GO terms with DEGs. The top 10 enriched terms with up- and down-regulated genes.

**C** The enriched molecular functions of GO terms with DEGs. The top 10 enriched terms with up- and down-regulated genes.

**
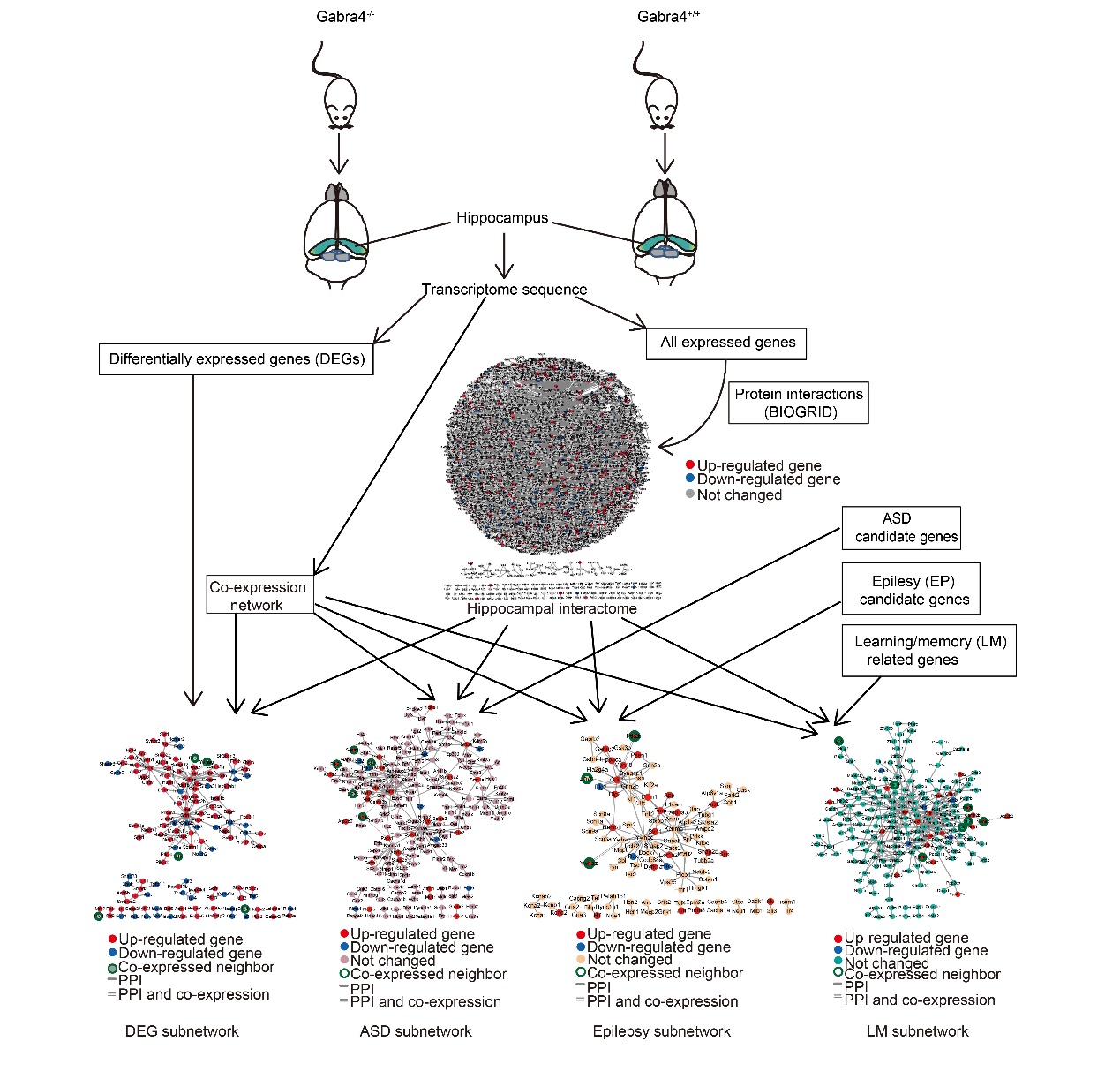
**

**Figure S5 Construction of hippocampal interactome network and extraction of DEG subnetwork, ASD subnetwork, LM subnetwork and EP subnetwork.**

The mouse hippocampal interactome, which contained 4,202 nodes and 9,205 edges, was constructed by integrating the hippocampal expressed genes and a protein interaction data from BioGRID (Biological General Repository for Interaction Datasets). Four subnetworks were extracted by mapping the “seeds” (DEGs, ASD candidate genes, LM-related genes or EP candidate genes) on to the hippocampal interactome network to include the interconnected “seeds” and their co-expressed neighbors in the hippocampal interactome network.


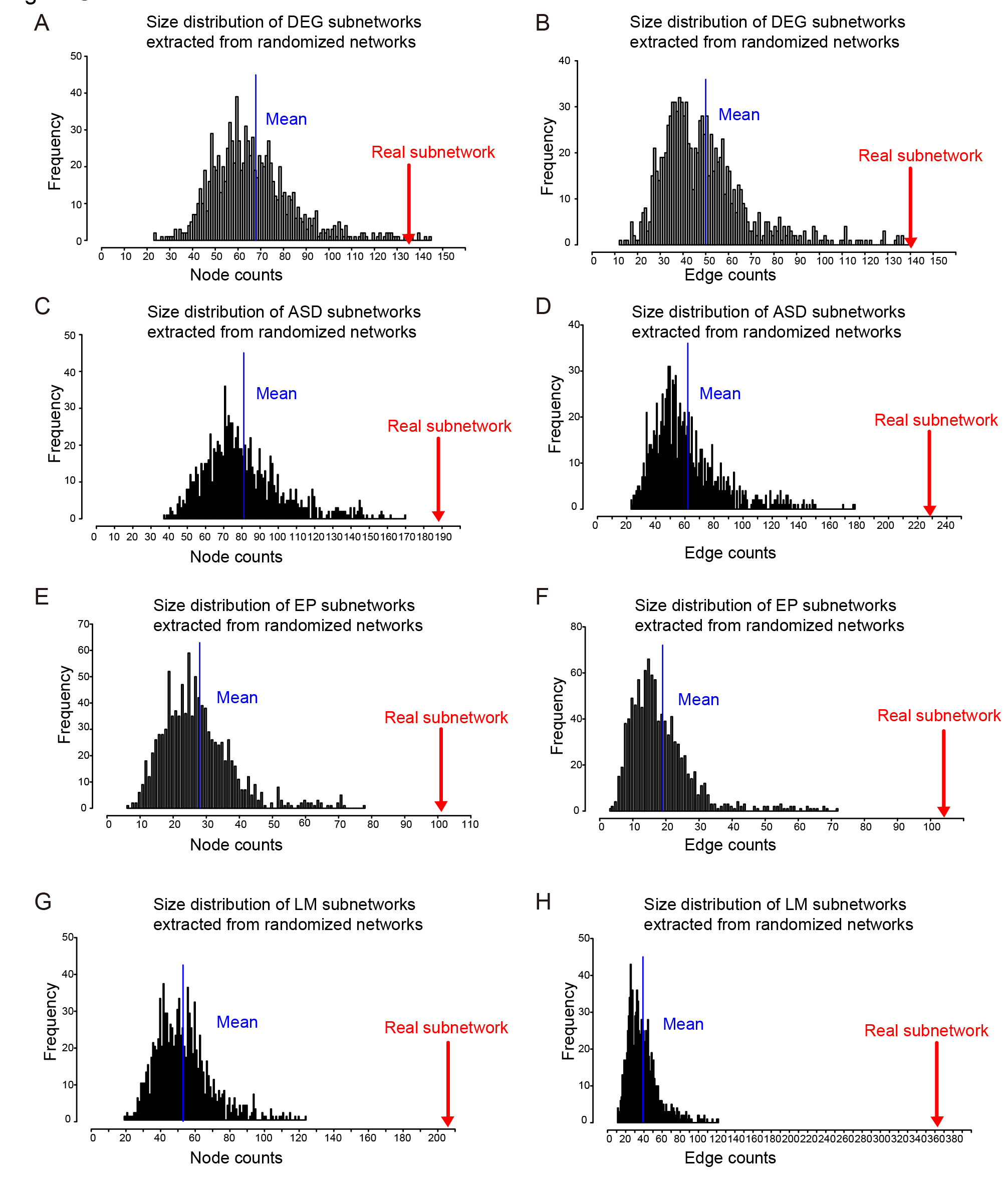


**Figure S6 Subnetworks extracted from randomized PPI networks compared to real subnetworks.**

Mouse PPI interactome from BioGRID was randomized for 1,000 times by shuffling nodes and 4 sets of seeds (DEGs, ASD candidates, EP candidates, LM candidates) were mapped onto the 1,000 randomized networks to extract subnetworks as controls. The size distribution of each control set of subnetworks was plotted in comparison with the size of each real subnetwork. Blue lines indicate the mean sizes of the control subnetworks, and red arrows indicate the sizes of the real subnetworks.

**A** Node counts distribution of DEG subnetworks extracted from randomized networks.

**B** Edge counts distribution of DEG subnetworks extracted from randomized networks.

**C** Node counts distribution of ASD subnetworks extracted from randomized networks.

**D** Edge counts distribution of ASD subnetworks extracted from randomized networks.

**E** Node counts distribution of EP subnetworks extracted from randomized networks.

**F** Edge counts distribution of EP subnetworks extracted from randomized networks.

**G** Node counts distribution of LM subnetworks extracted from randomized networks.

**H** Edge counts distribution of LM subnetworks extracted from randomized networks.

**
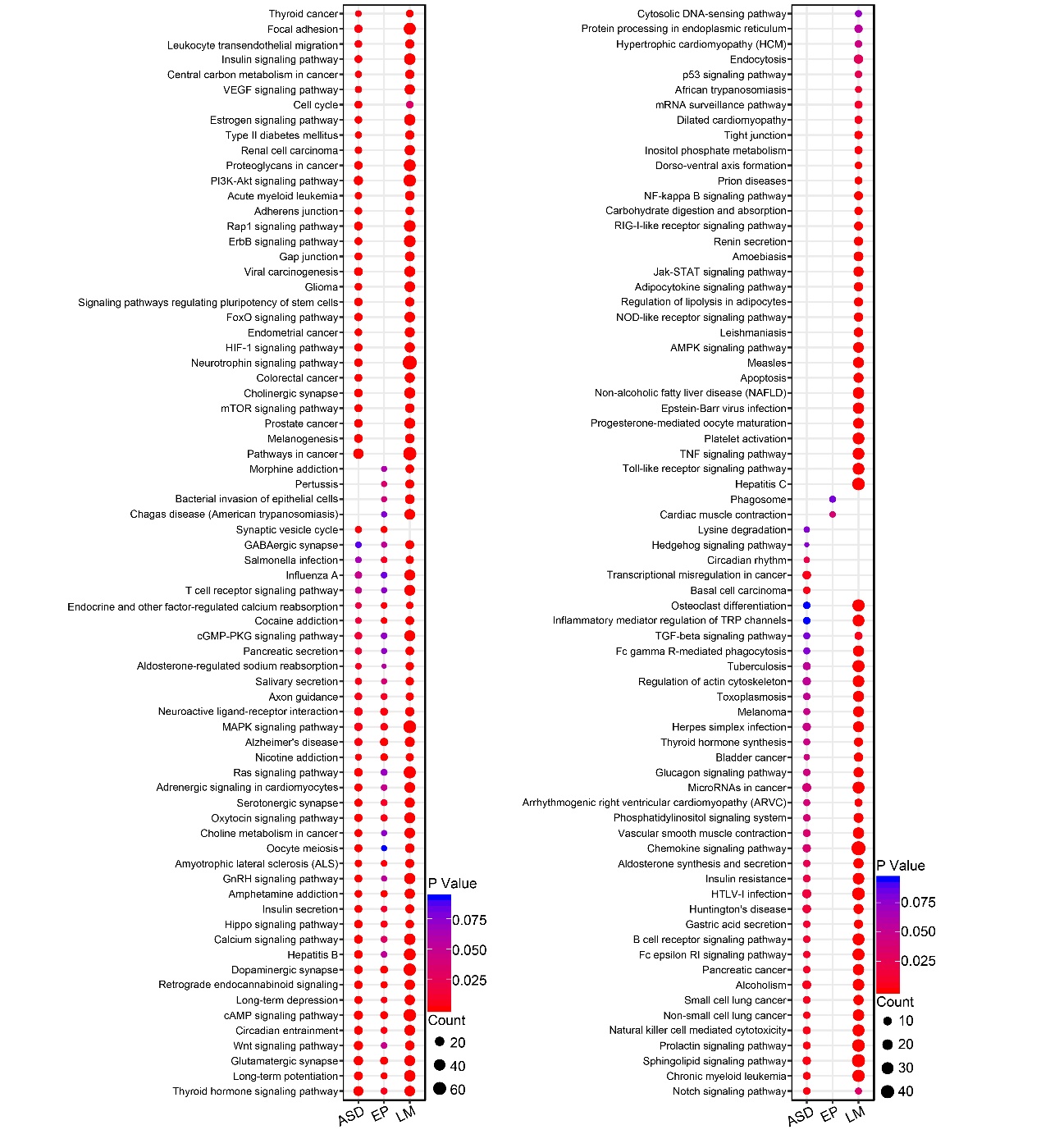
**

**Figure S7 Comparison of the enriched pathways with ASD, epilepsy and LM subnetworks.**

The comparison of enriched KEGG pathways with the three subnetworks. There are 37 enriched pathways shared by three subnetworks. The enrichment analysis was performed using DAVID bioinformatics tool with a *p*-value cutoff of 0.05 and FDR less than 0.05. ASD: autism; EP: epilepsy; LM: learning/memory.


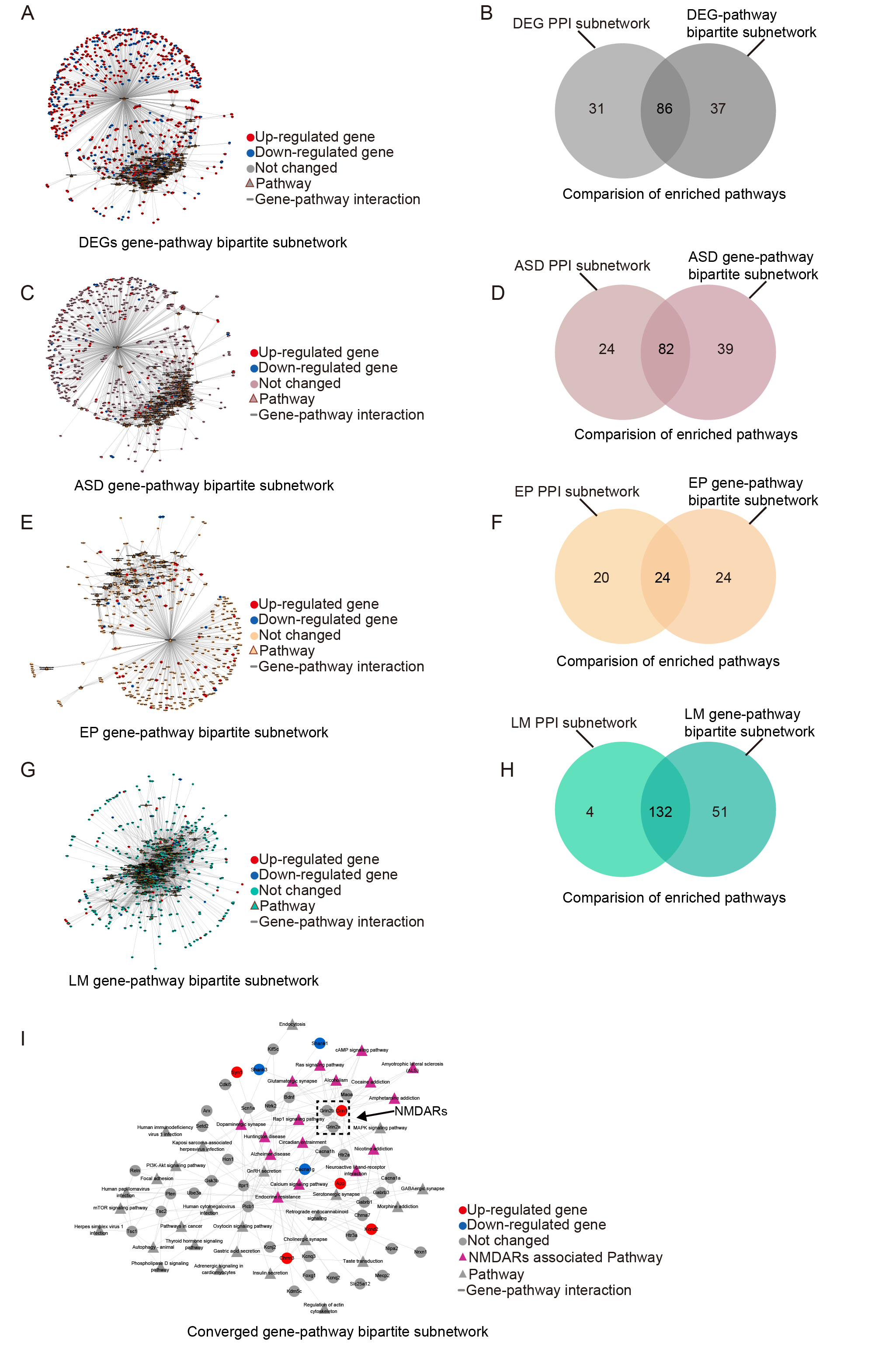


**Figure S8 Gene-pathway bipartite networks for DEGs, ASD candidates, EP candidates, LM-related genes.**

**A** DEG-pathway bipartite subnetwork. DEGs were mapped onto the hippocampal expressed gene-pathway bipartite network to extract a subnetwork including DEGs and their involved pathways, and the pathways significantly enriched with DEGs (*p* < 10^-6^) and containing at least 10 seed genes were kept.

**B** Comparison of enriched pathways in DEGs PPI subnetwork and DEG-pathway bipartite subnetwork.

**C** ASD gene-pathway bipartite subnetwork. ASD candidate genes were mapped onto the hippocampal expressed gene-pathway bipartite network to extract a subnetwork including ASD candidate genes and their involved pathways, and the pathways significantly enriched with ASD candidate genes (*p* < 10^-6^) and containing at least 10 seed genes were kept.

**D** Comparison of pathways enriched in ASD PPI subnetwork and ASD gene-pathway bipartite subnetwork.

**E** EP gene-pathway bipartite subnetwork. EP candidate genes were mapped onto the hippocampal expressed gene-pathway bipartite network to extract a subnetwork including EP candidate genes and their involved pathways, and the pathways significantly enriched with EP candidate genes (*p* < 10^-6^) and containing at least 10 seed genes were kept.

**F** Comparison of pathways involved in EP PPI subnetwork and EP gene-pathway bipartite subnetwork.

**G** LM gene-pathway bipartite subnetwork. LM-related genes were mapped onto the hippocampal expressed gene-pathway bipartite network to extract a subnetwork including LM-related genes and their involved pathways, and the pathways significantly enriched with LM-related genes (*p* < 10^-6^) and containing at least 10 seed genes were kept.

**H** Comparison of pathways involved in LM PPI subnetwork and LM gene-pathway bipartite subnetwork.

**I** The network module shared by all three gene-pathway bipartite subnetworks.

Fisher’s exact test was used for calculating all the *p* values.

**Tables**

The additional file 2 containing 31 sheets is uploaded separately.

**References**

1. Sung YH, Baek I-J, Kim DH, Jeon J, Lee J, Lee K, Jeong D, Kim J-S, Lee H-W: **Knockout mice created by TALEN-mediated gene targeting.** *Nature Biotechnology* 2013, **31:**23-24.

2. Yang M, Silverman JL, Crawley JN: **Automated three-chambered social approach task for mice.** *Curr Protoc Neurosci* 2011, **Chapter 8:**Unit 8 26.

3. Yang M, Zhodzishsky V, Crawley JN: **Social deficits in BTBR T+tf/J mice are unchanged by cross-fostering with C57BL/6J mothers.** *Int J Dev Neurosci* 2007, **25:**515-521.

4. Silverman JL, Yang M, Lord C, Crawley JN: **Behavioural phenotyping assays for mouse models of autism.** *Nat Rev Neurosci* 2010, **11:**490-502.

5. Kraeuter AK, Guest PC, Sarnyai Z: **The Open Field Test for Measuring Locomotor Activity and Anxiety-Like Behavior.** *Methods Mol Biol* 2019, **1916:**99-103.

6. Walf AA, Frye CA: **The use of the elevated plus maze as an assay of anxiety-related behavior in rodents.** *Nat Protoc* 2007, **2:**322-328.

7. Miedel CJ, Patton JM, Miedel AN, Miedel ES, Levenson JM: **Assessment of Spontaneous Alternation, Novel Object Recognition and Limb Clasping in Transgenic Mouse Models of Amyloid-beta and Tau Neuropathology.** *J Vis Exp* 2017.

8. Moore MD, Cushman J, Chandra D, Homanics GE, Olsen RW, Fanselow MS: **Trace and contextual fear conditioning is enhanced in mice lacking the alpha4 subunit of the GABA(A) receptor.** *Neurobiol Learn Mem* 2010, **93:**383-387.

9. Vorhees CV, Williams MT: **Morris water maze: procedures for assessing spatial and related forms of learning and memory.** *Nat Protoc* 2006, **1:**848-858.

10. Dobin A, Gingeras TR: **Mapping RNA-seq Reads with STAR.** *Curr Protoc Bioinformatics* 2015, **51:**11 14 11-19.

11. Chen YJ, Zhang M, Yin DM, Wen L, Ting AN, Wang P, Lu YS, Zhu XH, Li SJ, Wu CY, et al: **ErbB4 in parvalbumin-positive interneurons is critical for neuregulin 1 regulation of long-term potentiation.** *Proceedings Of the National Academy Of Sciences Of the United States Of America* 2010, **107:**21818-21823.
